# Clinical efficacy and identification of factors confer resistance to afatinib (tyrosine kinase inhibitor) in EGFR-overexpressing esophageal squamous cell carcinoma

**DOI:** 10.1038/s41392-024-01875-4

**Published:** 2024-06-28

**Authors:** Yanni Wang, Chang Liu, Huan Chen, Xi Jiao, Yujiao Wang, Yanshuo Cao, Jian Li, Xiaotian Zhang, Yu Sun, Na Zhuo, Fengxiao Dong, Mengting Gao, Fengyuan Wang, Liyuan Dong, Jifang Gong, Tianqi Sun, Wei Zhu, Henghui Zhang, Lin Shen, Zhihao Lu

**Affiliations:** 1 Key Laboratory of Carcinogenesis and Translational Research (Ministry of Education/Beijing), Department of Gastrointestinal Oncology, Peking University Cancer Hospital and Institute, Beijing, China; 2 Genecast Biotechnology Co., Ltd, Wuxi, PR China; 3 State Key Laboratory of Holistic Integrative Management of Gastrointestinal Cancers, Beijing Key Laboratory of Carcinogenesis and Translational Research, Department of Gastrointestinal Oncology, Peking University Cancer Hospital and Institute, Beijing, China; 4 Key Laboratory of Carcinogenesis and Translational Research (Ministry of Education), Department of Pathology, Peking University Cancer Hospital and Institute, Beijing, China; 5 Precision Scientific (Beijing) Co., Ltd., Beijing, China; 6 Generulor Company Bio-X Lab, Zhuhai, Guangdong, China; 7 Biomedical Innovation Center, Beijing Shijitan Hospital, Capital Medical University, Beijing, China; 8 Beijing Key Laboratory for Therapeutic Cancer Vaccines, Beijing Shijitan Hospital, Capital Medical University, Beijing, China

**Keywords:** Gastrointestinal cancer, Cancer therapy, Tumour biomarkers

## Abstract

Epidermal growth factor receptor (EGFR) is reportedly overexpressed in most esophageal squamous cell carcinoma (ESCC) patients, but anti-EGFR treatments offer limited survival benefits. Our preclinical data showed the promising antitumor activity of afatinib in EGFR-overexpressing ESCC. This proof-of-concept, phase II trial assessed the efficacy and safety of afatinib in pretreated metastatic ESCC patients (*n* = 41) with EGFR overexpression (NCT03940976). The study met its primary endpoint, with a confirmed objective response rate (ORR) of 39% in 38 efficacy-evaluable patients and a median overall survival of 7.8 months, with a manageable toxicity profile. Transcriptome analysis of pretreatment tumors revealed that neurotrophic receptor tyrosine kinase 2 (*NTRK2)* was negatively associated with afatinib sensitivity and might serve as a predictive biomarker, irrespective of EGFR expression. Notably, knocking down or inhibiting *NTRK2* sensitized ESCC cells to afatinib treatment. Our study provides novel findings on the molecular factors underlying afatinib resistance and indicates that afatinib has the potential to become an important treatment for metastatic ESCC patients.

## Introduction

Esophageal squamous cell carcinoma (ESCC) is one of the most common causes of cancer-related death worldwide.^1^ Treatment options for patients with metastatic ESCC have been limited in recent decades, and these patients have a 5-year survival rate < 5%.^2,3^ In combination with traditional chemotherapy, immunotherapy^4–6^ as a first-line treatment has shown promising efficacy; however, few options for second-line treatment have been identified. Therefore, there is still an urgent need to explore candidate treatment strategies for metastatic ESCC.

Epidermal growth factor receptor (EGFR) is known as a member of the ErbB family of receptor tyrosine kinases (RTKs). Upon binding with its ligand, EGFR undergoes a process of homodimerization or heterodimerization, followed by autophosphorylation. This in turn triggers several downstream effectors, such as the mitogen-activated protein kinase (MAPK)/extracellular signal-regulated kinase (ERK) and phosphatidylinositol 3-kinase (PI3K)/protein kinase B (AKT) signaling cascades, which play essential roles in regulating cellular proliferation and survival.^7,8^ EGFR overexpression is observed in 50%-70% of ESCC patients^9–12^ and is correlated with tumor invasion and poor survival,^13^ providing a rationale for anti-EGFR therapy in ESCC.

However, previous studies on anti-EGFR therapies, including cetuximab, panitumumab and gefitinib, have shown limited benefits for overall survival (OS),^14–17^ which may be partially attributed to the absence of patient selection strategies.^18,19^ Recently, squamous cell carcinoma (SCC) was shown to be enriched in EGFR, Erb-b2 receptor tyrosine kinase 2 (ErbB2) and ErbB3 with preferential dependencies,^20^ and high sensitivity to the pan-ErbB kinase inhibitor afatinib was also observed.^20^ However, a phase II study explored the efficacy of afatinib in platinum-resistant ESCC, and the objective response rate (ORR) was only 14.3%,^21^ which underscores the necessity of exploring biomarkers for determining the efficacy of afatinib. Our preclinical results showed that the antitumor activity of afatinib was superior to that of other EGFR inhibitors in ESCC, and EGFR overexpression was found to be a potential predictive biomarker for determining afatinib efficacy.^22^

Given this background, the current single-arm, proof-of-concept, phase II trial evaluated the efficacy and safety of afatinib in pretreated metastatic ESCC patients with EGFR overexpression (NCT03940976). Moreover, exploratory RNA sequencing (RNA-seq) was performed to investigate potential biomarkers of afatinib response and resistance. Additionally, in vitro and in vivo experiments were performed to dissect the underlying molecular mechanisms involved.

## Results

### Patients

**Fig. 1:**
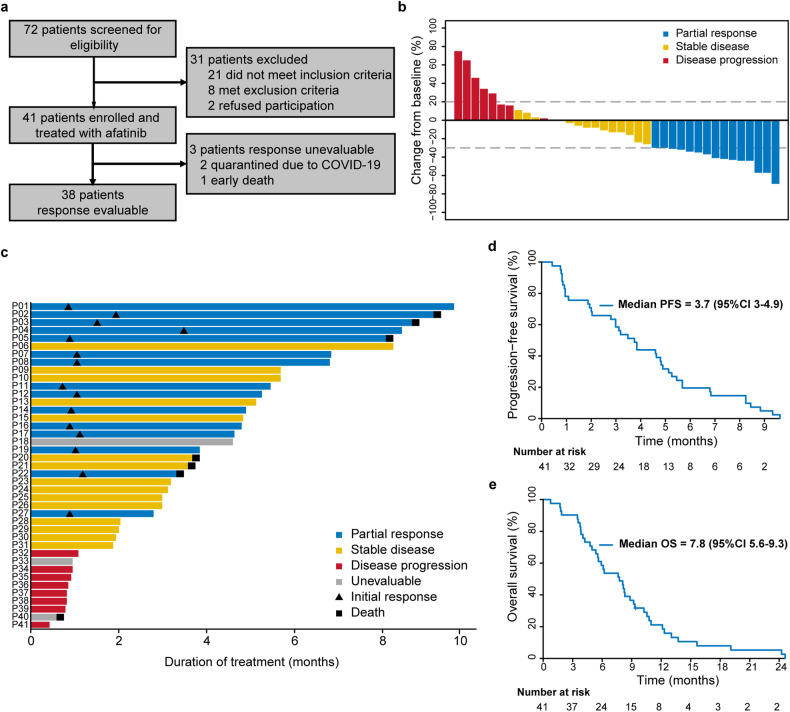
Study profile and antitumor activity of afatinib. **a** Study profile (*n* = 41). **b** Best percentage changes in target lesions from baseline (*n* = 38). **c** Treatment and response durations. The length of each bar represents the time from the first dose of afatinib to the time of the last radiographic assessment. **d** Kaplan‒Meier estimates of progression-free survival (PFS). **e** Kaplan‒Meier estimates of overall survival (OS)

Between May 15, 2019, and April 20, 2020, a total of 41 eligible patients with EGFR overexpression were enrolled in the study and treated with afatinib (Fig. 1a). All the patients were diagnosed with EGFR (3 + ) by IHC. Upon the cutpoint of the data collection (December 31, 2021), all the patients had developed disease progression, 40 individuals had died, and one participant was lost for subsequent follow-ups. The baseline and demographic clinical characteristics are listed in Table 1.

**Table 1:** Baseline characteristics

Variables	Total (*N* = 41)
Age (years), median (range)	61 (43–69)
Sex, *n* (%)	
Male	39 (95)
Female	2 (5)
ECOG performance status, *n* (%)	
0	1 (2)
1	37 (90)
2	3 (7)
Histologic grade, *n* (%)	
Well or moderately differentiated	25 (61)
Poorly differentiated	13 (32)
Unknown	3 (7)
Primary tumor location, *n* (%)	
Cervical	4 (10)
Upper thoracic	4 (10)
Middle thoracic	16 (39)
Lower thoracic	15 (37)
Esophagogastric junction (EGJ)	2 (5)
Site of metastases, *n* (%)	
Lymph node	35 (85)
Liver	16 (39)
Lung	16 (39)
Bone	11 (27)
Others^a^	12 (29)
Previous therapy, *n* (%)	
Surgery	16 (39)
Radiotherapy	21 (51)
Numbers of previous lines, *n* (%)	
1	33 (80)
> 1	8 (20)
Previous systemic therapies, *n* (%)	
Platinum	40 (98)
Taxanes	37 (90)
Anti-PD-1/PD-L1	8 (20)
Others^b^	7 (17)

^a^Kidney, adrenal, peritoneum and anastomosis. *ECOG* Eastern Cooperative Oncology Group

^b^Fluorouracil, irinotecan, tegafur/gimeracil/oteracil potassium, apatinib, nimotuzumab and cetuximab

### Antitumor activity

Among the 41 participants, 3 patients could not be evaluated for a radiological tumor response (Fig. 1a). The ORR of afatinib treatment was 39% (95% confidence interval (CI), 24–57) for the other 38 patients. All 15 responses were confirmed to be partial responses (PR), and 15 (39%) patients had stable disease (SD). The disease control rate (DCR) was 79% (95% CI, 63–90). The decrease in the target lesion burden from baseline is shown in Fig. 1b. The duration of afatinib treatment for 41 patients is shown in Fig. 1c. Among the 15 patients who exhibited an objective response, the median time to tumor response was 4.6 weeks (ranging from 3.1 to 15.1), and the median duration of response was 4.7 months (95% CI, 3.9–7.4). Four (27%) patients had a duration of response ≥ 6 months. The median progression-free survival (PFS) was 3.7 months (95% CI, 3–4.9; Fig. 1d). The median OS was 7.8 months (95% CI, 5.6–9.3; Fig. 1e).

### Adverse events

Treatment-related adverse events (TRAEs) are listed in Table 2. All the participants had one or more TRAEs, most commonly diarrhea (24 [59%]), rash or acne (24 [59%]), stomatitis (11 [27%]) or increased ALT/AST (8 [20%]). Grade 3 to 4 TRAEs occurred in 11 participants (27%), and no treatment-related deaths were reported. Two (5%) interstitial lung diseases were considered related to afatinib (grade 2 and grade 1). Nine (22%) participants had a reduction of dose to 30 mg/day, most commonly those with stomatitis (3 [7%]) and diarrhea (3 [7%]). Three patients experienced TRAEs leading to the discontinuation of afatinib, which were due to interstitial lung disease (grade 2), stomatitis (grade 3), or thrombocytopenia (grade 4).

**Table 2:** Adverse events

Adverse event (AE), No. (%)	Any Grade	Grade 1-2	Grade 3	Grade 4
Any event	41 (100)	30 (73)	10 (24)	1 (2)
Diarrhea	24 (59)	20 (49)	4 (10)	0 (0)
Rash or acne	24 (59)	23 (56)	1 (2)	0 (0)
Stomatitis	11 (27)	8 (20)	3 (7)	0 (0)
Increased ALT/AST	8 (20)	6 (15)	2 (5)	0 (0)
Dry skin	7 (17)	7 (17)	0 (0)	0 (0)
Paronychia	5 (12)	5 (12)	0 (0)	0 (0)
Epistaxis	4 (10)	4 (10)	0 (0)	0 (0)
Fatigue	3 (7)	3 (7)	0 (0)	0 (0)
Pruritus	2 (5)	2 (5)	0 (0)	0 (0)
Interstitial lung disease	2 (5)	2 (5)	0 (0)	0 (0)
Thrombocytopenia	2 (5)	1 (2)	0 (0)	1 (2)
Muscle spasms	1 (2)	1 (2)	0 (0)	0 (0)

### The response to afatinib is associated with the MAPK pathway

**Fig. 2:**
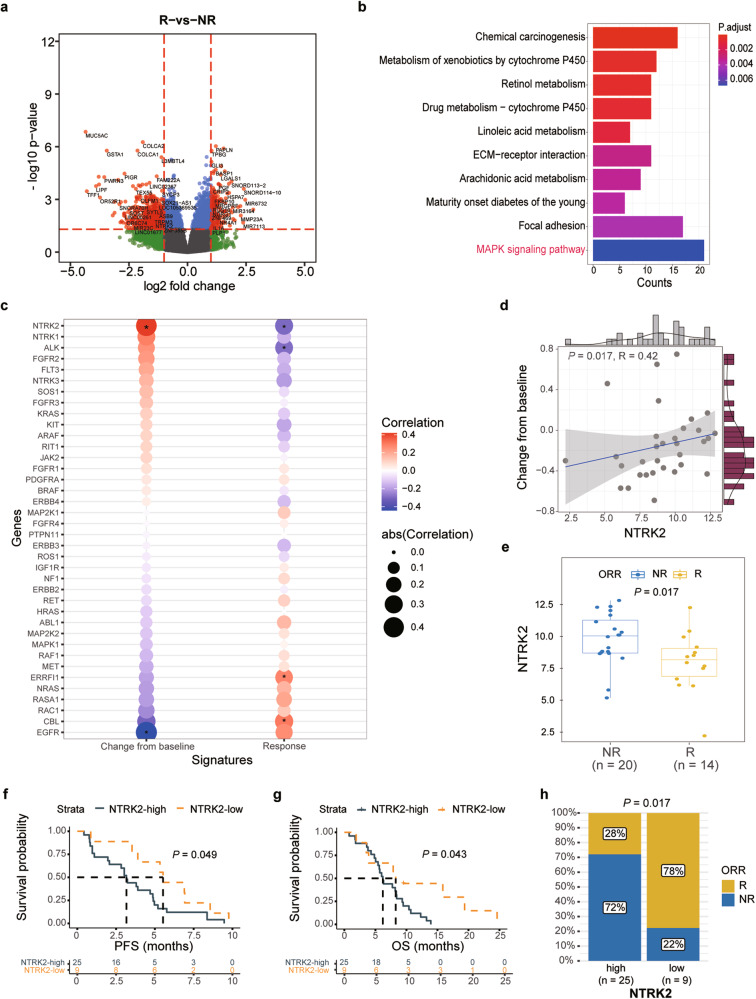
*NTRK2* expression correlates with afatinib activity in patients with metastatic ESCC. **a** The volcano plot shows the up- and downregulated differentially expressed genes (DEGs) between responders (R) and nonresponders (NR, including patients with SD or PD as the best response). Genes with *P*-values < 0.05 and a |log2 (-fold change)| > 1 (as revealed by DESeq2) were considered differentially expressed. The x-axis represents the log2-fold change (FC), and the y-axis represents the log10 *P* value. **b** All candidate DEGs were subjected to pathway enrichment analysis using the enrich KEGG function of the R Cluster Profiler. The enriched KEGG terms (*P* < 0.01) are shown in the bar plot. **c** The correlation of RTK/MAPK/RAS gene expression (normalized read counts) with changes from baseline or best response (PD, SD, PR: levels 1–3). The colored circles represent the correlation coefficient (r) between pairs of these variables and the change in tumor size (Spearman correlation) or response level (Kendall’s rank correlation). The asterisk inside the colored circles indicates the statistical significance of the pairwise correlation. **d** Two-sided Spearman correlations between *NTRK2* expression and changes from baseline. **e** Differences in the *NTRK2* expression level between responders (R, *n* = 14) and nonresponders (NR, *n* = 20) (two-sided Wilcoxon test). PFS (**f**) and OS (**g**) curves were estimated by the Kaplan–Meier method. *NTRK2* expression levels were dichotomized by using the maximally selected rank statistics (‘maxstat’) method for OS benefit (*NTRK2* high: normalized read count > 7.95). (mPFS: 5.5 *versus* 3.2 months, *P* = 0.049; mOS: 8.3 *versus* 6.2 months, *P* = 0.043). **h** The ORR in the *NTRK2* high- and low-expression groups (ORR: 78% *versus* 28%, Fisher’s exact test *P* = 0.017)

To identify potential mechanisms of afatinib response in EGFR-overexpressing ESCC, we subjected all available pretreatment biopsy samples to RNA-seq analysis (*n* = 34). We identified 175 upregulated genes and 593 downregulated genes in afatinib responders (*n* = 14) compared to nonresponders (*n* = 20) (Fig. 2a and Supplementary Fig. 1a). The 768 differentially expressed genes (DEGs) were used for subsequent KEGG pathway enrichment analysis, which revealed important roles for several pathways, such as MAPK signaling, extracellular matrix (ECM)-receptor interaction, and focal adhesion (adjusted *P* value < 0.05; Fig. 2b).

It was previously suggested that dysregulation of alternative RTKs or activation of downstream MAPK pathway components are the mechanisms by which cells can compensate for EGFR tyrosine kinase inhibitor (TKI) treatment.^23^ We thus assessed how the mRNA levels of genes in the RTK/MAPK pathway correlated with tumor reduction or the best response (PR, SD, or PD). Interestingly, we found that the *EGFR* mRNA expression level was significantly associated with a reduction in tumor size (R = −0.44, *P* = 0.011; Fig. 2c and Supplementary Fig. 1b) and afatinib response (*P* = 0.039; Supplementary Fig. 1c), suggesting that a relatively high expression of *EGFR* might contribute to the afatinib response in patients with EGFR-overexpressing ESCC. In contrast, we found that high expression levels of several genes, including neurotrophic receptor tyrosine kinase 2 (*NTRK2*), *NTRK1* and anaplastic lymphoma kinase (*ALK*), were associated with an increase in tumor size (Fig. 2c). Among these genes, *NTRK2* significantly correlated with increased tumor size (R = 0.42, *P* = 0.017; Fig. 2c, d) and decreased afatinib response (*P* = 0.017; Fig. 2e), suggesting that the *NTRK2* mRNA expression level negatively correlates with afatinib sensitivity. Both *NTRK2* and *EGFR* were significantly associated with the response rate, with area under the curve (AUC) values of 0.743 (95% CI, 0.643-0.850) and 0.711 (95% CI, 0.571-0.900), respectively (Supplementary Fig. 1d, e).

To assess whether these two potential biomarkers could predict survival prognosis, we identified the best cutoff point for both variables by using the maximally selected rank statistics (‘maxstat’) method for dividing patients into subgroups with high versus low levels. Indeed, when the best cutoff point was used for OS, the *NTRK2*-low subpopulation had longer median PFS (5.5 *versus* 3.2 months, *P* = 0.049; Fig. 2f) and OS (8.3 *versus* 6.2 months, *P* = 0.043; Fig. 2g) and a greater response rate (ORR: 78% *versus* 28%, Fisher’s exact test *P* = 0.017; Fig. 2h) than did the *NTRK2*-high subgroup. The opposite trends were observed for *EGFR* expression (Supplementary Figs. 1f–h); high *EGFR* expression was correlated with improved median PFS (5.4 *versus* 3.2 months, *P* = 0.049; Supplementary Fig. 1f) and OS (8.1 *versus* 6.1 months, *P* = 0.044; Supplementary Fig. 1g) and a greater response rate (ORR: 80% *versus* 25%, Fisher’s exact test *P* = 0.006; Supplementary Fig. 1h) than low *EGFR* expression was. Furthermore, we conducted a combined biomarker analysis of patients with high/low *NTRK2* and *EGFR* expression to analyze differences in OS, PFS, and ORR. Our findings indicated that the OS and PFS were longest in the *NTRK2*^low^_*EGFR*^high^ group, with no significant variations observed in OS and PFS among the other three groups (Supplementary Figs. 1i, j). The *NTRK2*^low^_*EGFR*^high^ group also exhibited the highest proportion of ORR (100%) (Supplementary Fig. 1k). These data indicate that the combined use of *NTRK2* and *EGFR* biomarkers may enhance the precision of patient stratification. Our data indicate that *NTRK2* and *EGFR* expression are potential molecular determinants of afatinib response. Given that afatinib is an irreversible EGFR-TKI, the *EGFR* expression level may affect the afatinib response via a direct mechanism.

### *NTRK2* markedly reduced sensitivity to afatinib, irrespective of *EGFR* expression

**Fig. 3:**
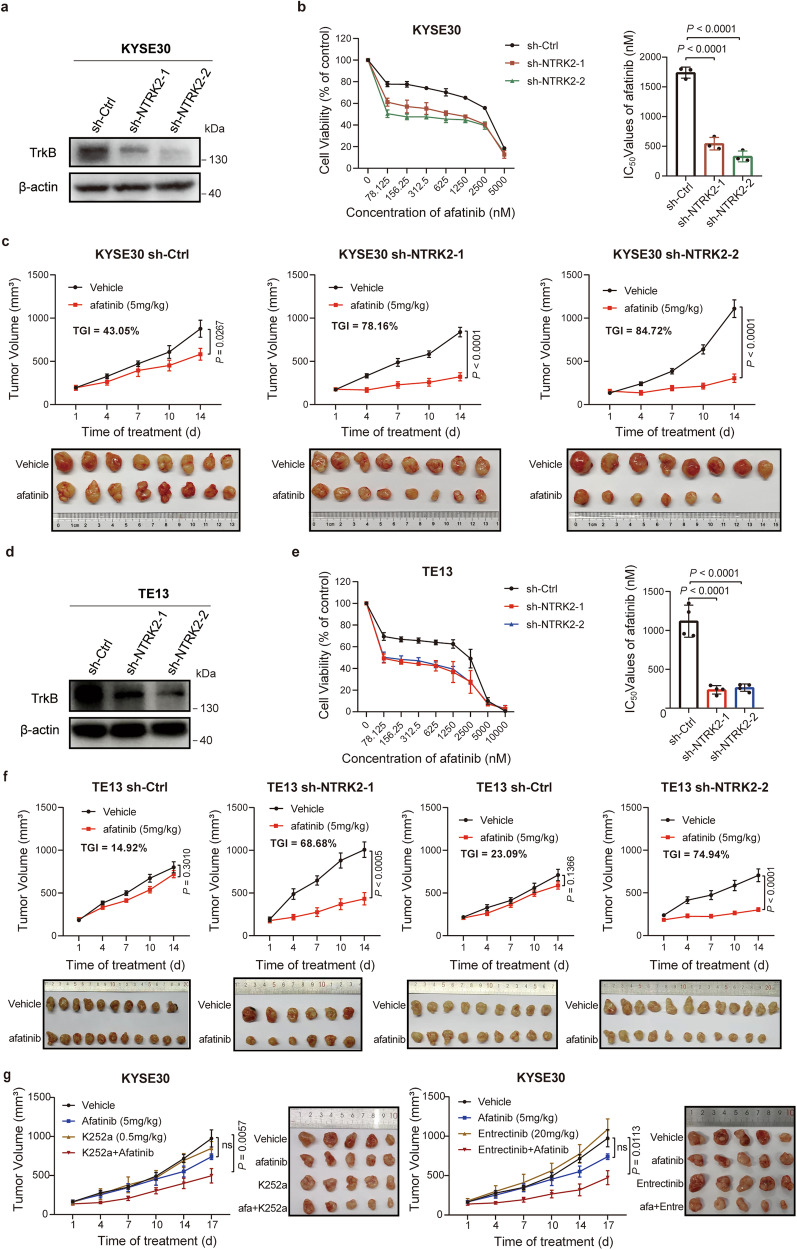
Knockdown of *NTRK2* (TrkB) enhances ESCC sensitivity to afatinib. **a** Western blotting showing the expression of the TrkB protein or β-actin in KYSE30 sh-Ctrl cells or upon knockdown of *NTRK2* (two different shRNAs, sh-*NTRK2*-1 and sh-*NTRK2*-2, were used). **b** Proliferation of KYSE30 cells with sh-Ctrl or the indicated shRNAs after treatment with increasing concentrations of afatinib for 72 h is shown (left), and the IC_50_ values of the three cell lines were evaluated by a CCK-8 assay (right). The data are presented as the mean ± s.d. of three independent experiments. **c** KYSE30 cells transduced with sh-*Ctrl* or sh-*NTRK2* were subcutaneously injected into mice and then treated with afatinib or saline (vehicle). Tumor growth curves (top) and representative images (bottom) were shown. (sh-*Ctrl* and sh-*NTRK2*-1: *n* = 8 per group; sh-*NTRK2*-2: vehicle, *n* = 8; afatinib, *n* = 6). The data are presented as the mean ± s.e.m. **d** Western blot analysis of TrkB expression in TE13 cells transduced with lentivirus encoding an *NTRK2*-specific or nontargeting (ctrl) shRNA. β-Actin was used as a loading control. **e** *NTRK2*-knockdown TE13 cells (sh-*NTRK2*) and control TE13 cells (sh-*Ctrl*) were treated with a range of concentrations of afatinib for 72 h, after which CCK-8 assays were performed to assess cell viability (left) and IC_50_ values (right). The data are presented as the mean ± s.d. of four independent experiments. **f** Growth curves (top) and representative images (bottom) of TE13 tumors from sh-*Ctrl* + vehicle (*n* = 10), sh-*Ctrl* + afatinib (*n* = 11), sh-*NTRK2*-1 + vehicle (*n* = 6) and sh-*NTRK2*-1 + afatinib (*n* = 6) -treated nude mice; and growth curves (top) and representative images (bottom) of TE13 tumors from sh-*Ctrl* + vehicle (*n* = 9), sh-*Ctrl* + afatinib (*n* = 9), sh-*NTRK2*-2 + vehicle (*n* = 12) and sh-*NTRK2*-2 + afatinib (*n* = 11)- treated NOD/SCID mice. **g** The efficacy of the pan-Trk inhibitors K252a (left) or Entrectinib (right) was assessed when administered alone or in combination with afatinib in an afatinib-resistant model (KYSE30) (*n* = 5 mice per group). Representative tumor images were shown. The data are presented as the mean ± s.e.m. ns, not significant. *P* values were calculated using one-way ANOVA or unpaired two-tailed *t*-tests. TGI, tumor growth inhibition. The experiments were repeated three times independently with similar results; the data from one representative experiment are shown (**a**, **d**)

To explore the underlying mechanisms by which afatinib resistance may occur in *NTRK2*-overexpressing patients. We first performed in vitro and in vivo experiments to explore how *NTRK2* inhibition may impact afatinib sensitivity. We measured the afatinib IC_50_ values for five ESCC cell lines (EC109, KYSE450, KYSE30, TE13 and TE1). As shown in Supplementary Fig. 2a, afatinib had limited antiproliferative effects on KYSE30, TE13 and TE1 cells. Notably, a strong increase in the expression of tyrosine receptor kinase B (TrkB, encoded by *NTRK2*) was observed in resistant cells, including KYSE30, TE13 and TE1 cells (Supplementary Fig. 2b), and this effect was not influenced by the level of *EGFR* expression, indicating that *NTRK2* may function as a more effective biomarker for predicting afatinib treatment response than does *EGFR*. Later, we explored whether *NTRK2* downregulation improved afatinib sensitivity in two different cell lines (Fig. 3a–f), KYSE30 (with both high *EGFR* and *NTRK2* expression) and TE13 (with low *EGFR* expression and high *NTRK2* expression), by applying a short hairpin RNA (shRNA) strategy. As expected, shRNA targeting *NTRK2* markedly reduced the afatinib IC_50_ in both cell lines (Fig. 3a, b, d, e). In another afatinib-resistant cell line, TE1 (a cell line with low *EGFR* and high *NTRK2* levels), similar findings were observed, as *NTRK2* silencing drastically decreased the IC_50_ value of afatinib (*P* = 0.0016; Supplementary Fig. 4a). We then investigated the impact of *NTRK2* overexpression on afatinib-sensitive cell lines using lentiviral-mediated techniques. As illustrated in Supplementary Fig. 5, *NTRK2* expression was significantly increased in EC109 cells following transfection with LV-*NTRK2* (OE-*NTRK2*) compared to the control (OE-*NC*). CCK-8 analysis revealed that upregulation of *NTRK2* led to enhanced cell viability upon afatinib treatment and higher IC_50_ values for afatinib in EC109 cells (*P* = 0.003).

To validate whether *NTRK2* influences afatinib sensitivity in vivo, we used *NTRK2*-silenced KYSE30 or TE13 cells and control cells to establish xenograft models in immunodeficient mice. Compared to vehicle, afatinib markedly repressed the growth of KYSE30 sh-*NTRK2* cell-originating xenografts by 78.16% (*P* < 0.0001) and 84.72% (*P* < 0.0001), respectively, while it inhibited sh-*Ctrl* tumor xenograft growth by only 43.05% (*P* = 0.0267) (Fig. 3c). We then examined the antitumor activity of afatinib in TE13sh-*Ctrl* and TE13sh-*NTRK2* xenografts. The growth of sh-*NTRK2* tumors was significantly inhibited by afatinib compared with that of vehicle-treated mice (tumor growth inhibition [TGI]: 68.68%–74.94%, *P* < 0.0005 and *P* < 0.0001, respectively). However, the inhibitory effects of afatinib on the sh-*Ctrl* model were weak (TGI: 14.92%-23.09%, *P* = 0.3010 and *P* = 0.1366; Fig. 3f). Taken together, the data above indicate that low *NTRK2* expression may function as an effective predictor of the afatinib response, irrespective of *EGFR* expression.

Then, we sought to investigate whether the combination of NTRK inhibitors could increase the sensitivity to afatinib in ESCC cell lines. As illustrated in Supplementary Fig. 6, our results demonstrated that the combination of pan-Trk inhibitors (Entrectinib and K252a) significantly increased the inhibitory impact of afatinib on KYSE30 and TE13 cells. We investigated the impact of two inhibitors on the growth of xenografts derived from KYSE30 cells. Figure 3g demonstrates that the combination of afatinib with either K252a or Entrectinib significantly inhibited xenograft growth, surpassing the efficacy of any single drug treatment. Through cellular and animal experiments, we observed that the combined treatment of afatinib with pan-Trk inhibitors exhibited a greater inhibitory effect on cells or xenografts than afatinib alone.

### High *NTRK2* expression confers resistance to afatinib through the MAPK/ERK pathway

**Fig. 4:**
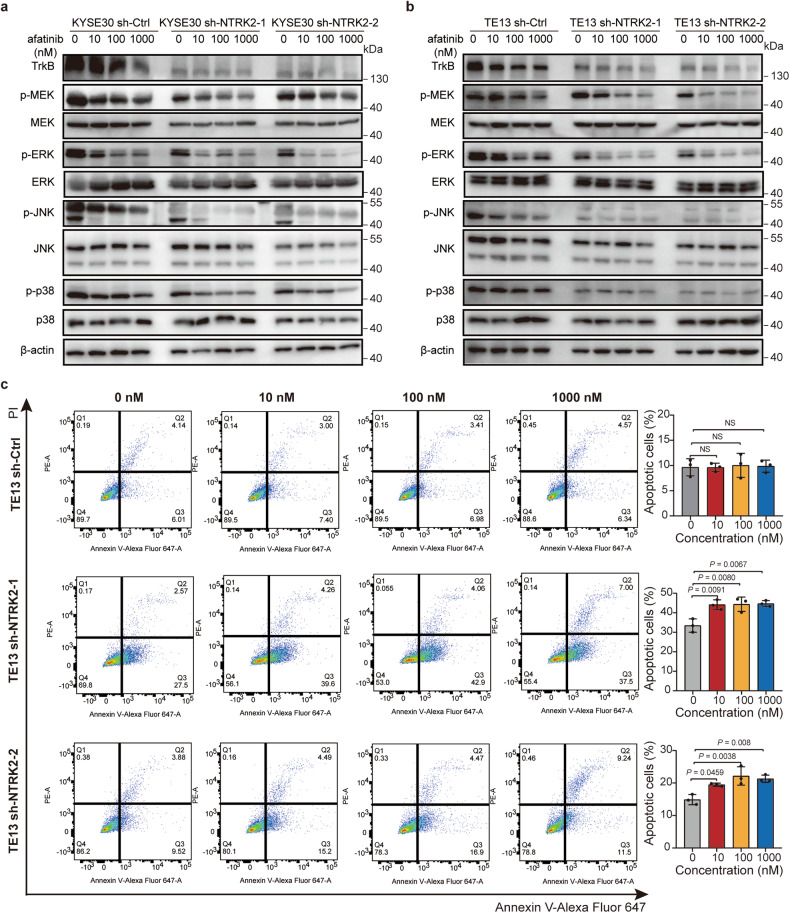
*NTRK2* knockdown enhances the efficacy of afatinib through the MAPK/ERK signaling pathway. **a** Western blotting analyses of MAPK/ERK pathway targets in KYSE30 cells (**a**) and TE13 cells (**b**) transfected with sh-*Ctrl* or sh-*NTRK2* and treated for 48 h with afatinib at 10 nM, 100 nM or 1000 nM. **c** FACS analyses of Annexin V-Alexa Fluor 647- and PI-stained TE13 cells after knockdown of the indicated shRNAs or sh-Ctrl for 48 h with afatinib at 10 nM, 100 nM and 1000 nM (left) and quantification of apoptosis (right). All the quantitative bar data are presented as the means ± s.d. of three individual experiments. NS, not significant. *P*-values were calculated using one-way ANOVA. Representative images are shown

To determine the underlying molecular mechanisms by which low *NTRK2* expression confers afatinib sensitivity in ESCC cells, we analyzed alterations in the cell cycle, apoptosis and essential signaling cascades. Cell cycle analysis revealed that afatinib did not induce G0/G1 arrest in sh-*Ctrl* or sh-*NTRK2* TE13 cells (Supplementary Fig. 3), while G0/G1 arrest was evident in sh-*NTRK2* TE1 cells compared to sh-*Ctrl* cells (Supplementary Fig. 4c). Moreover, we investigated apoptosis in TE13 and TE1 cell lines. In TE13 and TE1 cells with *NTRK2* suppression, extensive apoptosis was detected at concentrations of 10 nM and 100 nM, whereas we only observed a weak induction in apoptosis in TE13 and TE1 cells harboring sh-*Ctrl* at a concentration of 1000 nM (Fig. 4c and Supplementary Fig. 4d). The concentration required to completely inhibit cell cycle arrest and apoptosis was significantly lower for cells expressing low *NTRK2* levels than for cells expressing high *NTRK2* levels.

We also analyzed the expression patterns of key players in the MAPK pathway, which is a critical pathway involved in patient response to afatinib treatment, as described above. Consistent with these findings, in both KYSE30 and TE13 cells, the expression of phosphorylated MEK (p-MEK), ERK (p-ERK), JNK (p-JNK) and p38 (p-p38) were significantly inhibited by *NTRK2* knockdown after afatinib treatment with a low concentration (Fig. 4a, b). Similarly, *NTRK2* suppression reduced the levels of p-MEK, p-ERK, p-JNK and p-p38 in TE1 cells in the presence of low concentrations of afatinib (Supplementary Fig. 4b). The above data suggest that *NTRK2* may confer resistance to afatinib by mediating the activation of the MAPK/ERK signaling pathway.

## Discussion

To the best of our knowledge, this is the first study to assess the efficacy and safety of afatinib in ≥ 2^nd^ line treatment for ESCC patients with EGFR overexpression. Afatinib was well tolerated and had a promising response rate (ORR 39%) (meeting the predetermined response criterion [25%]) and a median OS of 7.8 months. Exploratory RNA-seq analysis revealed that *NTRK2* expression was negatively correlated with the afatinib response. Notably, *NTRK2* knockdown sensitized ESCC cells to afatinib treatment both in vitro and in vivo. Combination therapy of afatinib and TrkB inhibitors exerted a synergistic suppressive effect on an afatinib-resistant model. Mechanistically, *NTRK2* inhibition may attenuate resistance to afatinib, possibly by suppressing MAPK/ERK signaling.

According to prior clinical reports, the response rates to current second-line treatments for ESCC are relatively poor (3-20.2%).^19,24^ Several previous trials have tried to target EGFR in unselected patient populations and found low response rates.^18,19,21,25^ Therefore, the objective response rate and the median PFS recorded in the present study are promising compared with those in other trials involving unselected advanced ESCC patients. However, more than 60% of the metastatic ESCC patients with EGFR overexpression were still nonresponsive, suggesting that there are other underlying mechanisms in addition to EGFR itself.

Exploratory biomarker analysis based on RNA-seq also revealed a candidate biomarker, *NTRK2*, which was negatively correlated with afatinib sensitivity. The predictive value of *NTRK2* gene expression tends to be relatively greater than that of *EGFR*, as evidenced by the AUC values. Further in vitro experiments revealed that cells expressing elevated *NTRK2* had decreased sensitivity to afatinib, even though there was variability in *EGFR* expression. Moreover, *NTRK2* knockdown attenuated resistance to afatinib in two *NTRK2*-overexpressing cell lines in vitro and in vivo, irrespective of *EGFR* expression, possibly through cell cycle arrest and apoptosis induction. Our data strongly demonstrated that *NTRK2* was a key factor in afatinib resistance in ESCC patients. The tropomyosin receptor kinase TrkB, encoded by the *NTRK2* gene, plays a crucial role in the development of the nervous system.^26^ It has been indicated in tumor formation and progress across different types of cancer.^27–29^ The overexpression of TrkB significantly correlated with a poor clinical prognosis in various human malignancies.^30–32^ Aberrations in the TRK pathway, such as gene fusions and overexpression, activate downstream signaling cascades via RAS/MAPK/Erk or PI3K/Akt pathways, thus initiating oncogenic processes.^33^ However, studies investigating *NTRK2* expression and related therapeutics in ESCC are limited. Several findings suggest that high expression of *NTRK2* is directly correlated to the resistance to anti-EGFR therapy in CRC^34^ and cisplatin- and 5-fluorouracil-treated ESCC^35^ at the cellular level. Here, we present the novel finding that high *NTRK2* expression is related to resistance to afatinib, and we subsequently explored the potential mechanism of *NTRK2*-mediated resistance to afatinib.

Mechanistically, the growth inhibitory effect of *NTRK2* knockdown is related to the reduced phosphorylation of MEK (p-MEK), ERK (p-ERK), JNK (p-JNK) and p38 (p-p38). Therefore, *NTRK2* might lead to afatinib resistance by activating the MAPK/ERK signaling pathway. Previous studies revealed that activation of bypass survival pathways via other RTKs or via MAPK/ERK or PI3K/AKT signaling may trigger EGFR-TKI resistance in several other cancers,^23,36–38^ such as amplification of MET,^36^ overexpression of hepatocyte growth factor (*HGF*)^37^ and loss of *PTEN*.^38^ Although substantial progress has been made in dissecting the mechanisms of resistance to EGFR TKIs, our results reveal the new discovery that the NTRK2-MAPK/ERK signaling axis confers EGFR-TKI resistance in ESCC.

Previous studies have demonstrated that there is crosstalk between EGFR and TrkB (*NTRK2*).^34,39–41^ TrkB could be transactivated via EGFR, promoting its signaling responsiveness, which is important for neuronal cells to their final position.^39^ In ovarian cancer cells, EGFR and TrkB activation cooperate and stimulate cell proliferation and cell survival.^40^ Whereas the synergy effect of EGFR and TrkB inhibitors appear to be cell-line specific in squamous cell carcinoma (SCC) cell lines,^41^ implying further refinement is required in selecting patient subgroups for this treatment. Based on preclinical data presented here, the combined administration of afatinib and TrkB inhibitors demonstrated a synergistic suppressive effect on an afatinib-resistant model. These findings suggest that combining a TrkB inhibitor with afatinib might be a novel treatment strategy to overcome afatinib resistance in ESCC patients.

Our study has several limitations. First, the sample size was small, and future verification should be conducted in a prospective multicenter study. Second, while our study identified *NTRK2* as a potential predictive biomarker and therapeutic target, further exploration in a clinical setting is warranted to validate its efficacy and applicability in patients. Consequently, an ongoing prospective clinical trial is investigating the use of afatinib in ESCC patients who exhibit low *NTRK2* expression (NCT05818982).

In conclusion, afatinib has revealed the promising effect in pretreated metastatic ESCC patients with EGFR overexpression, and its adverse event profile is manageable. Furthermore, our study revealed that high *NTRK2* expression leads to afatinib resistance through the MAPK/ERK pathway, independent of *EGFR* expression levels. Additionally, the combined administration of afatinib and TrkB inhibitors exhibited a synergistic suppressive effect on an afatinib-resistant model. These findings may provide novel insights into the molecular factors contributing to afatinib resistance and suggest that afatinib represents an important treatment strategy for metastatic ESCC. Notably, refining patient selection and implementing rational targeted therapies will likely further enhance survival outcomes for patients with metastatic ESCC.

## Materials and methods

### Study design and patients

This was an open-label, single-arm, phase II trial performed at Peking University Cancer Hospital & Institute. The key eligibility criteria included: (1) histologically confirmed ESCC with EGFR (3 + ) staining; (2) disease progression after one or more prior lines of systemic therapy; (3) 18 to 70 years of age; (4) at least one measurable tumor lesion per Response Evaluation Criteria in Solid Tumors, version 1.1 (RECIST v1.1); (5) Eastern Cooperative Oncology Group (ECOG) performance status (PS) of 0-2; (6) estimated life expectancy of more than 3 months; (7) adequate organ function. The key exclusion criterion was a history of interstitial lung disease. Considering efficacy biomarkers as one of the secondary endpoints, we collected pretreatment formalin-fixed paraffin-embedded (FFPE) tumor samples from each participant. Full list of inclusion/exclusion criteria could be found in the Supplementary Information.

This study and the tissue sample collection were approved by the Clinical Research Ethics Committee of Peking University Cancer Hospital and Institute Consent (2019YJZ18). The study was conducted in compliance with the Helsinki Declaration and its later amendments or comparable ethical standards. Written informed consent was obtained from each participant before participation and were informed of the sample collection methods used for translational studies before they provided signed consent.

### Treatment and assessments

Patients received 40 mg afatinib orally once daily. If patients occurred any grade 3 or higher drug-related adverse events, grade 2 diarrhea lasting 48 h or more, or rash or acne for 7 consecutive days or more, then afatinib was given until the patient recovered to grade 1 or less, with a 10 mg decrease in dose to a minimum dose of 20 mg. The primary endpoint was the objective response rate (ORR). The secondary endpoints included the disease control rate (DCR), adverse events (AEs), progression-free survival (PFS) and overall survival (OS). Tumor assessments were performed once at 4 and 8 weeks postdose and once every 8 weeks thereafter until disease progression (defined according to RECIST v1.1), patient withdrawal, unacceptable toxicity, or investigator decision. The responses were categorized as complete response (CR), partial response (PR), stable disease (SD), or progressive disease (PD) according to RECIST v1.1. The ORR was defined as the proportion of patients who experienced CR or PR as the best overall response. The DCR was defined as the proportion of patients who experienced CR, PR or SD as the best overall response. AEs were collected according to the National Cancer Institute Common Terminology Criteria for Adverse Events (NCI-CTCAE) version 4.0. PFS was calculated from the first dose of afatinib to radiological disease progression or death from any cause. OS was calculated as the time from the first dose to death from any cause.

### EGFR expression

We assessed EGFR protein expression using immunohistochemistry (IHC) based on the manufacturer’s instructions (Clone E30, DAKO, Glostrup, Denmark; 1:50). Positive staining was defined: any membrane staining above the background level (defined as the level noted in a negative control sample) in ≥10% of cancer cells of any intensity, with 1+ indicating faint or barely perceptible membrane staining, 2+ indicating weak to moderate staining of the complete cell membrane, and 3+ indicating strong staining of the complete cell membrane. The staining was evaluated by two blinded investigators independently.

### RNA sequencing and transcriptome profiling

FFPE tumor tissue sections collected prior to afatinib treatment were processed for RNA isolation using the RNeasy FFPE extraction kit (QIAGEN, Inc., Germany) according to the manufacturer’s instructions. RNA concentrations were measured using the Qubit RNA HS Assay Kit (Thermo Scientific, USA), and RNA integrity was determined employing the Labchip GX Touch HT method (PerkinElmer, USA). An aliquot of 50 ng of FFPE RNA was used as the input for library construction using a SMARTer Stranded Total RNA-Seq Kit ver. 2 (Takara Bio, Inc., Japan). After reverse transcription, the cDNAs were ligated with index adapters, and the rRNAs were removed using an R-probe. All libraries were enriched over 13 cycles using a SimpliAmp Thermal Cycler (Thermo Fisher). The yields were measured using Qubit dsDNA HS Assay kits (Thermo Fisher), and the peak library distributions were assessed employing high-sensitivity DNA kits (Agilent, USA). The libraries were sequenced (150 PE) on an Illumina NovaSeq 6000 platform (Illumina, USA). Sequencing adapters and low-quality bases were removed with Trimmomatic (version 0.32), and the sequences were mapped to the human reference genome (hg19) using the subread package with the default parameters. To quantify gene expression, the FeatureCounts routine of Subread was used to summarize the read counts against the UCSC RefSeq genes, and DESeq2 was subsequently used for read count normalization and differential gene expression (DEG) analysis. The DEG criteria were *P* < 0.05 and |log2 (-fold change) | > 1. Pathway enrichment analysis of candidate DEGs was performed using the enrichKEGG function of the R package Cluster Profiler with a *P* cutoff < 0.01. The expression of genes in the RTK/MAPK/RAS pathway is shown as the normalized read count according to DESeq2. The correlation of RTK/MAPK/RAS gene expression (normalized read counts) with changes in tumor size or best response (PD, SD, PR: levels 1-3) was analyzed by using Spearman correlation or Kendall’s rank correlation, respectively.

### Antibodies and drugs

The anti-TrkB (ab18987) antibody was purchased from Abcam (USA). Anti-EGFR (no. 4267), anti-MEK (no. 4694), anti-p-MEK (no. 2338), anti-ERK1/2 (no. 4695), anti-p-ERK1/2 (no. 4370), anti-JNK (no. 9252), anti-p-JNK (no. 4668), anti-p38 (no. 8690) and anti-p-p38 (no. 4511) were purchased from Cell Signaling Technology (USA). Antibodies against β-actin (A5441) was obtained from Sigma-Aldrich, USA. Afatinib (BIBW2992) dimaleate (S7810) was obtained from Selleck Chemical (USA) and dissolved in normal saline. The pan-Trk inhibitors Entrectinib (S7998) and K252a (HY-N6732) were obtained from Selleck Chemicals (USA) and MedChem Express, respectively. The drugs were dissolved in dimethyl sulfoxide (D2650, Sigma).

### Cell lines

The cell lines EC109, KYSE450, KYSE30, TE13 and TE1 were obtained from the National Infrastructure of Cell Line Resource (Beijing, China). All cells were sub-cultured in RPMI 1640 medium (Gibco, USA) or DMEM (Gibco) added with 10% FBS (Gibco) and 1% penicillin-streptomycin (Gibco) at 37 °C in a 5% CO_2_ humidified incubator.

### Establishment of knockdown cell lines

The lentiviral vectors harboring sh-*Ctrl* and sh-*NTRK2* were purchased from GeneChem (Beijing, China) and used to transfect cells according to the manufacturer’s instructions. Briefly, 1 × 10^5^ cells/well were transfected with the indicated lentiviruses for 48 h in the presence of 5 μg/mL polybrene (Yeasen, USA). The transfected cells were selected by growth in 2–4 μg/mL puromycin (InvivoGen, USA) for 2 weeks. Western blotting was used to assess transfection efficiency. The shRNA sequences used were as follows:

sh-*Ctrl*: TTCTCCGAACGTGTCACGT;

sh-*NTRK2*-1: TGGGATGTTGGTAACCTGGTT;

sh-*NTRK2*-2: GCAGGTGATCCGGTTCCTAAT.

### Establishment of a stable cell line expressing *NTRK2*

Lentiviral particles overexpressing *NTRK2* (vector control group: OE-*NC*; overexpression group: OE-*NTRK2*) were amplified and sequenced by HanBio Technology (Shanghai, China). EC109 cells were then transfected with *NTRK2* overexpression lentiviral vectors to induce *NTRK2* overexpression. Transfection was carried out using a transfection reagent containing polybrene (5 μg/mL) for 24 h, followed by a 2-week selection period with puromycin (2 μg/mL).

### Cell viability assay and IC_50_ estimation

Cell Counting Kit-8 (CCK-8) (CK04, Dojindo, Japan) assays were used to assess cell viability. Cells were seeded into 96-well plates at 2 to 3 × 10^4^/mL and treated with afatinib. After 72 h treatment, CCK-8 solution was added and the mixture was incubated for 2 h before measuring the absorbance at 450 nm. The IC_50_ value was used to measure the relative cytotoxicity via GraphPad software.

### Cell cycle and apoptosis assays

For the cell cycle assay, the cells were harvested and fixed in 75% ethanol overnight at 4 °C and washed with 1 × phosphate buffered saline (PBS). Subsequently, the cells were stained with propidium iodide (BD Pharmingen, Belgium) for 30 min at room temperature in the dark. Apoptotic cells were labeled using an Annexin V-Alexa Fluor 647/PI kit (P04D12, Gene-Protein Link, China). Analysis was performed with flow cytometry, and the data were imported from the instrument to ModFit LT 4.0 and FlowJo 10.4.0 software.

### Western blotting

Total proteins were extracted from cells using RIPA buffer and protease inhibitor cocktail tablets (Roche), followed by incubation on ice 30 min and centrifugation at 12,000 rpm for 12 min at 4 °C. A protease/phosphatase inhibitor (4906845001, Roche, USA) was included in all experiments aimed at investigating protein phosphorylation. Protein concentrations were determined using a BCA Protein Assay Kit (Beyotime Biotechnology, China). Subsequently, 30–40 µg of protein was loaded onto gels. The proteins were transferred to polyvinylidene difluoride membranes (Bio-Rad) using a Bio-Rad wet transfer system. The membranes were blocked in 5% skim milk or BSA for 1 h at room temperature. They were incubated with primary antibodies diluted in universal antibody diluent (WB500D, NCM Biotech) at 4 °C. The next day, the membranes were cleaned and exposed to horseradish peroxidase-conjugated secondary antibodies (CST, USA), then washed and treated with Western Lightning (P90720, MILLIPORE). The blots were visualized using the Amersham ImageQuant 800 system (GE, USA) following to the manufacturer’s instructions.

### Animal experiments

All studies with mice were approved by the Ethics Committee of Animal Experiments of Peking University Cancer Hospital (approval number ECEA 2021-11). 6-week-old female nonobese diabetic/severe combined immunodeficiency (NOD/SCID) mice or BALB/c nude mice were purchased from Vital River (Beijing, China). KYSE30, KYSE30 sh-*Ctrl*, KYSE30 sh-*NTRK2*, TE13 sh-*Ctrl* and TE13 sh-*NTRK2* cells were resuspended in PBS and Corning Matrigel at a 1:4 ratio and then injected (5 × 10^6^ cells per injection) into the flank. The mice in each group were randomized and treated when the tumor volume reached approximately 200 mm^3^. The mice received the following treatments: vehicle (normal saline or 100 μl of corn oil daily by oral gavage), afatinib (5 mg/kg daily by oral gavage), entrectinib (20 mg/kg daily by oral gavage) and K252a (0.5 mg/kg every other day by intraperitoneal injection). Tumor sizes were measured twice a week, and the tumor volume (TV) was calculated according to the standard formula: length × width × width ×0.5. Tumor growth inhibition (TGI) was defined as follows: 1- [(TV_f_ –TV_i_) _treated_ / (TV_f_ –TV_i_) _control_] ×100% (subscripts f and i refer to the final and initial values, respectively).

### Statistical analysis

Simon’s minimax two-stage design (Simon, 1989) was used to estimate the required sample size. The null hypothesis was an ORR ≤ 10%, and the alternative hypothesis was an ORR ≥ 25%. The type I error rate was 0.05, and the power was 0.8. In stage I, 22 patients were enrolled. The following contingencies were in place: If there were 0-2 responses, the study was to cease. Otherwise, an additional 18 patients were enrolled (total sample size 40). The null hypothesis was rejected if 8 or more responses were noted.

All analyses were performed using R ver. 3.6.1 or GraphPad Prism ver. 8.0 software. Efficacy analysis included all patients with measurable disease at baseline who underwent at least one posttreatment assessment. The safety analysis included all patients who received at least one dose of afatinib. The 95% confidence intervals (CIs) of the ORR and DCR were calculated using the Clopper-Pearson method. The Kaplan‒Meier method was utilized to estimate PFS, OS, and DOR. The maximally selected rank statistics (‘maxstat’) method was employed to dichotomize the two potential biomarkers for survival benefit using the survminer R package. The data are expressed as the mean ± s.d. or s.e.m. of three or more individual experiments. Differences among groups were evaluated using Student’s *t* test or one-way analysis of variance (ANOVA). *P* values < 0.05 was considered statistically significant.

### Supplementary information

Supplementary materials

Trial Protocol

## Data Availability

RNA sequencing data have been deposited into the figshare database (DOI: 10.6084/m9.figshare.23664765). All the data supporting the findings of this study can be obtained from the corresponding author upon reasonable request.
