# Supplementary material for: Clinical efficacy and identification of factors confer resistance to afatinib (tyrosine kinase inhibitor) in EGFR-overexpressing esophageal squamous cell carcinoma
Source: Signal Transduct Target Ther. 2024 Jun 28;9:153. doi: 10.1038/s41392-024-01875-4 (PMC11211462; doi:10.1038/s41392-024-01875-4)
Supplement: Supplementary file 1 — Supplementary materials [file 41392_2024_1875_MOESM1_ESM.docx]

Supplementary Materials for

**Clinical efficacy and identification of factors confer resistance to afatinib (tyrosine kinase inhibitor) in EGFR-overexpressing esophageal squamous cell carcinoma**

Yanni Wang^1#^, Chang Liu^1#^, Huan Chen^2#^, Xi Jiao^1^, Yujiao Wang^1^, Yanshuo Cao^1^, Jian Li^3^, Xiaotian Zhang^3^, Yu Sun^4^, Na Zhuo^1^, Fengxiao Dong^1^, Mengting Gao^1^, Fengyuan Wang^1^, Liyuan Dong^1^, Jifang Gong^3^, Tianqi Sun^5^, Wei Zhu^6^, Henghui Zhang^7,8^**^*^**, Lin Shen^3^**^*^** and Zhihao Lu^1*^

^1^Key Laboratory of Carcinogenesis and Translational Research (Ministry of Education/Beijing), Department of Gastrointestinal Oncology, Peking University Cancer Hospital and Institute, Beijing, China.

^2^Genecast Biotechnology Co., Ltd, Wuxi, P.R. China.

^3^State Key Laboratory of Holistic Integrative Management of Gastrointestinal Cancers, Beijing Key Laboratory of Carcinogenesis and Translational Research, Department of Gastrointestinal Oncology, Peking University Cancer Hospital and Institute, Beijing, China.

^4^Key laboratory of Carcinogenesis and Translational Research (Ministry of Education), Department of Pathology, Peking University Cancer Hospital and Institute, Beijing, China.

^5^Precision Scientific (Beijing) Co., Ltd., Beijing, China.

^6^Generulor Company Bio-X Lab, Zhuhai, Guangdong, China.

^7^Biomedical Innovation Center, Beijing Shijitan Hospital, Capital Medical University, Beijing, China.

^8^Beijing Key Laboratory for Therapeutic Cancer Vaccines, Beijing Shijitan Hospital, Capital Medical University, Beijing, China.

**^#^These authors contributed equally to this work.**

**^*^Correspondence to:**

Henghui Zhang, Tie-Yi Road 10, Hai-Dian District, Beijing 100038, China. Tel: +86-10-63925015. Email: zhhbao@ccmu.edu.cn.

Lin Shen, Fu-Cheng Road 52, Hai-Dian District, Beijing 100142, China. Tel: +86-10-88196561; Fax: +86-10-88196561; Email: shenlin@bjmu.edu.cn.

Zhihao Lu, Fu-Cheng Road 52, Hai-Dian District, Beijing 100142, China. Tel: +86-10-88196561; Email: [zhihaolupku@bjmu.edu.cn](mailto:zhihaolupku@bjmu.edu.cn)

**This PDF file includes:**

Supplementary Text

Figures. S1 to S6

**Supplementary Figures**

**
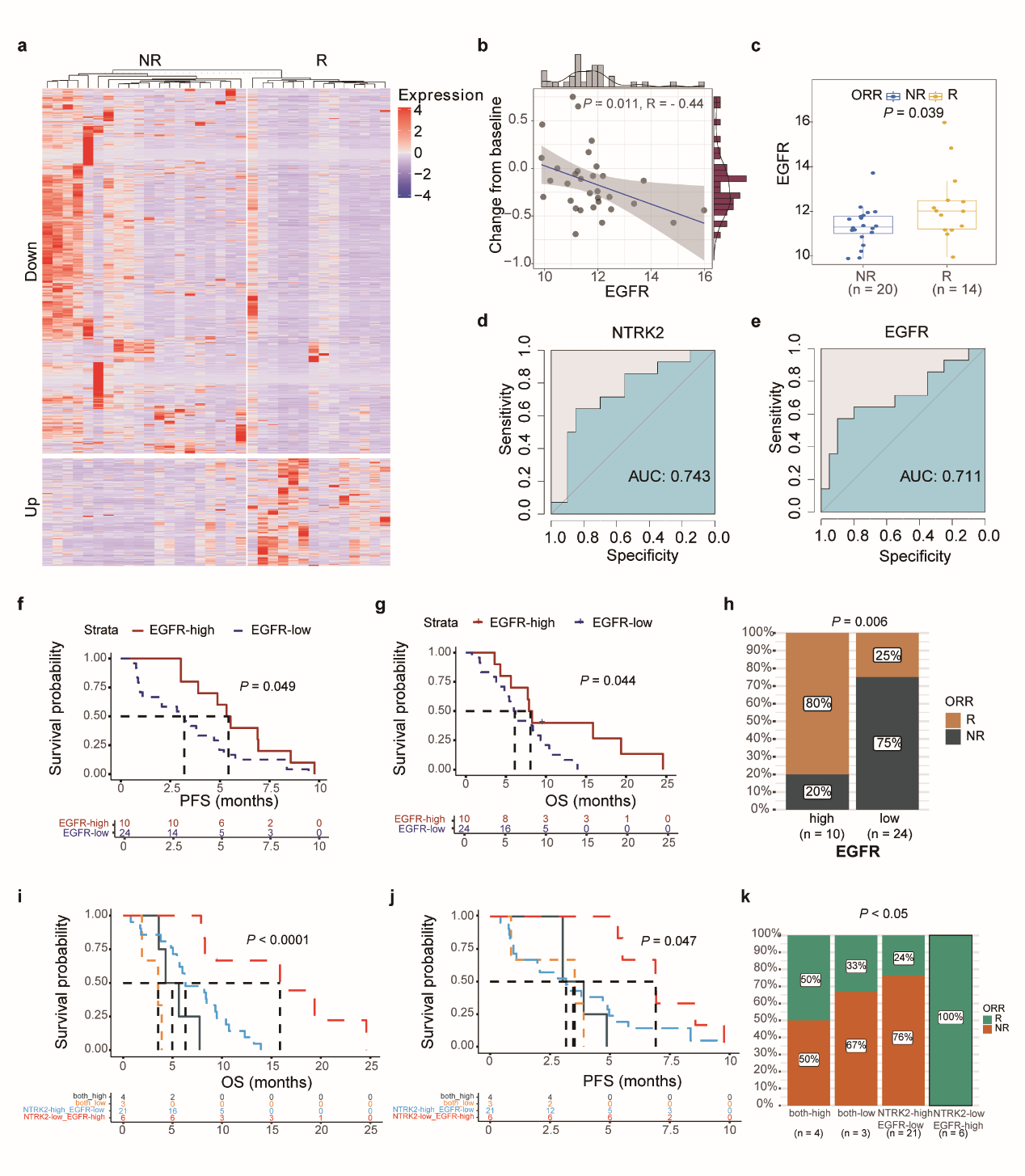
**

**Supplementary Fig. 1 *NTRK2* and *EGFR* expression are potential molecular determinants of the afatinib response. a** Heatmap showing the up- and down-regulated differentially expressed genes (DEGs) between responders (R) and nonresponders (NR). Genes with P values < 0.05 and a |log2 (-fold change)| > 1 were considered differentially expressed. **b** Two-sided Spearman correlations between *EGFR* expression and changes from baseline. **c** Differences in the *EGFR* expression level between responders (R) and nonresponders (NR, two-sided Wilcoxon test). Receiver operating characteristic (ROC) curve for the prediction of response based on the expression level (normalized read counts) of the *NTRK2* **d** or *EGFR* **e** gene measured by RNA-seq. PFS **f** and OS **g** curves were estimated by the Kaplan–Meier method. *EGFR* expression levels were dichotomized by using the maximally selected rank statistics (‘maxstat’) method for OS benefit (EGFR high: normalized read count > 11.983). (mPFS: 5.4 *versus* 3.2 months, *P* = 0.049; mOS: 8.1 *versus* 6.1 months, *P* = 0.044). **h** Association of *EGFR* expression with ORR after afatinib treatment (ORR: 80% *versus* 25%, Fisher's exact test *P* = 0.006). Kaplan‒Meier plots for OS **i** and PFS **j** from afatinib treatment in patients stratified by EGFR and NTRK2 expression. (OS, *P* < 0.0001; PFS, *P* = 0.047). **k** The ORR differed significantly in both the EGFR and NTRK2 expression groups (*P* < 0.05). Patients were classified into four subtypes; *EGFR*-low_*NTRK2*-low, *EGFR*-low_*NTRK2*-high, *EGFR*-high_*NTRK2*-low, and *EGFR*-high_*NTRK2*-high.


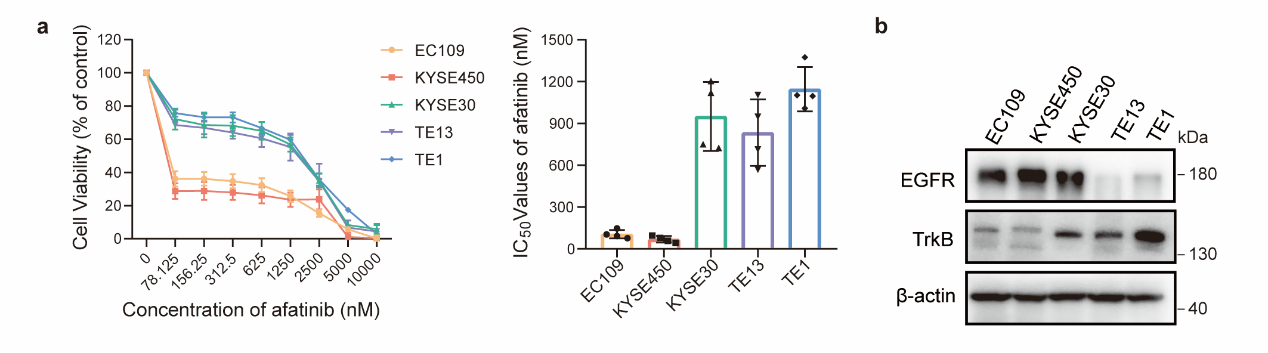


**Supplementary Fig. 2 Afatinib inhibited cell proliferation in ESCC cells with low *NTRK2* expression.** **a** CCK-8 assay data showing the viability of EC109, KYSE450, KYSE30, TE13 and TE1 cells treated (72 h) with a range of concentrations of afatinib (left) and the IC_50_ values (right). The data are presented as the mean ± s.d. (n = 4 individual experiments). **b** Western blotting revealing TrkB and EGFR levels in all five cell lines. An antibody against β-actin was used as a loading control. The experiments were repeated three times independently with similar results; the data from one representative experiment are shown.


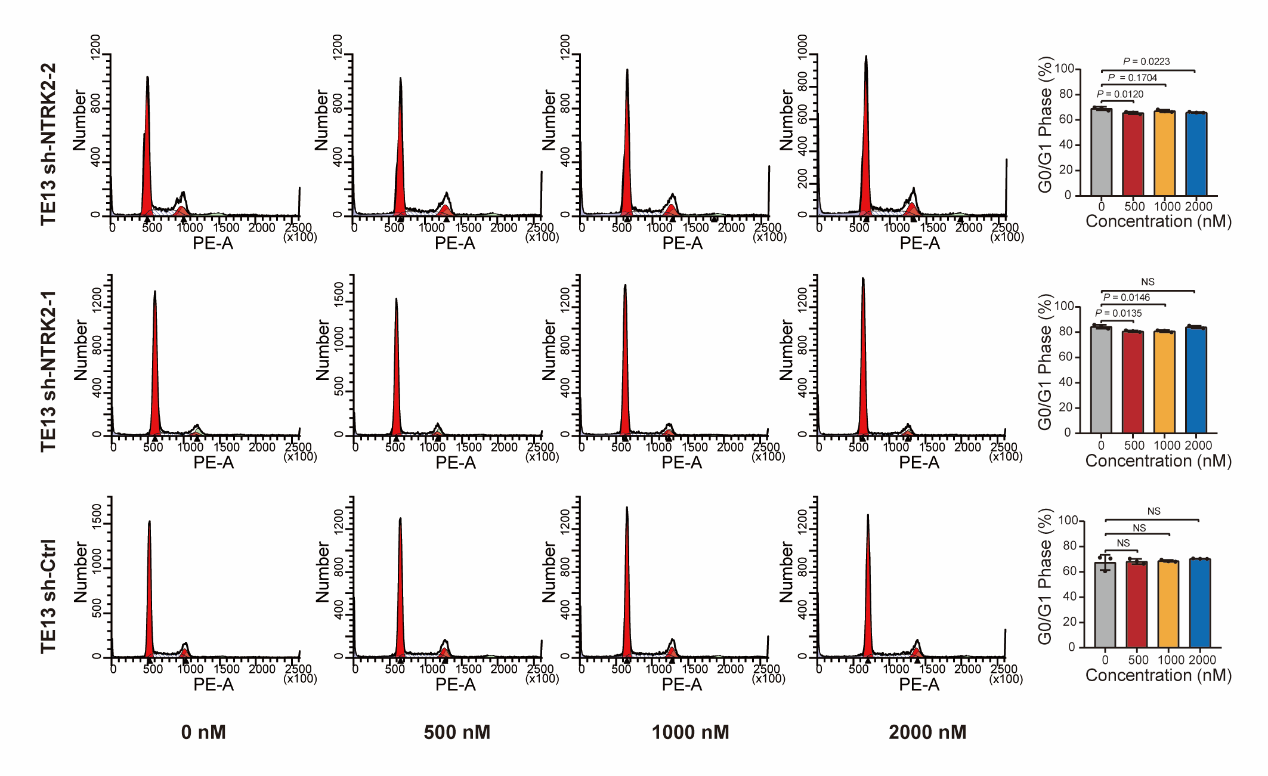


**Supplementary Fig. 3 Effect of *NTRK2* on the cell cycle in the TE13 cell line.** Cell cycle analysis of sh-*Ctrl*- and sh-*NTRK2*-transfected TE13 cells after treatment with afatinib at 500 nM, 1,000 nM or 2,000 nM was performed by flow cytometry. The portion of cells in each phase is shown (left), and the quantification of the G0/G1 phase fractions is shown (right). The data are shown as the mean ± s.d. *P* values were calculated using one-way ANOVA. The experiments were repeated three times independently with similar results; the data from one representative experiment are shown.


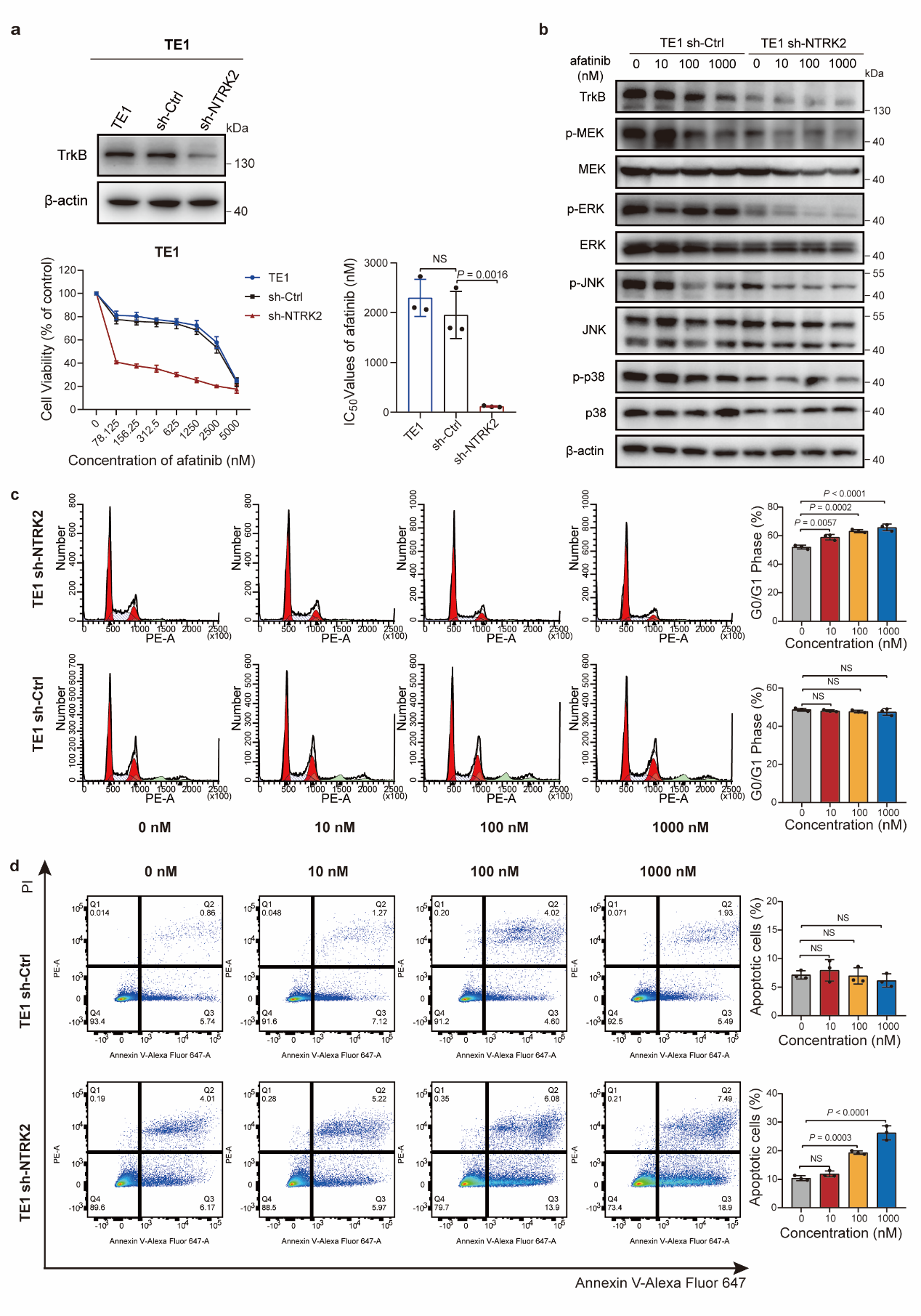


**Supplementary Fig. 4 *NTRK2* knockdown enhances the efficacy of afatinib through the MAPK/ERK signaling pathway in TE1 cells. a** Cell viability and IC_50_ data for TE1 cells transfected with sh-*NTRK2* or sh-*Ctrl* detected by CCK-8 assays after treatment with afatinib (0-1,000 nM) for 72 hours. The data are presented as the mean ± s.d. of three independent experiments. **b** Western blot assays were used to evaluate the total protein levels of MEK, ERK, JNK, and p38 and the phosphorylation levels of MEK, ERK, JNK, and p38 in transfected TE1 cells (sh-*Ctrl*: negative control shRNA; sh-*NTRK2*: shRNA against *NTRK2*). **c** Representative cell cycle analysis of *NTRK2*-knockdown TE1 cells (sh-*NTRK2*) and control TE1 cells (sh-*Ctrl*) (left) and quantification of cells in the G0/G1 phase (right). The data are presented as the mean ± s.d. of triplicate wells from a representative experiment. **d** Percentage of apoptotic cells measured by flow cytometry after treatment with afatinib (0 nM, 10 nM, 100 nM or 1,000 nM) for 48 hours in sh-*Ctrl*- and sh-*NTRK2*-transfected TE1 cells. The data are presented as the mean ± s.d. (n = 3 independently treated cell cultures). *P* values were calculated using one-way ANOVA or unpaired two-tailed t tests. Western blotting was repeated twice independently with similar results. Representative images are shown.


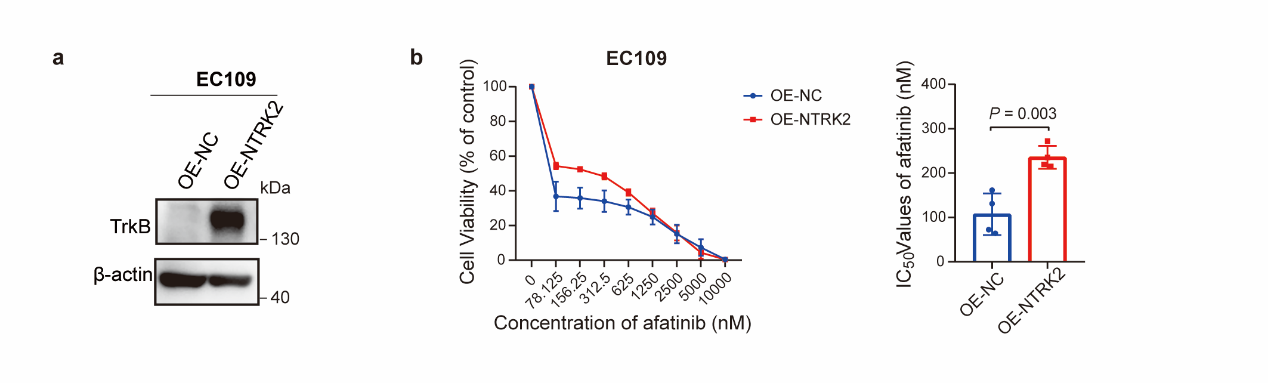


**Supplementary Fig. 5 Overexpression of *NTRK2* confers resistance in afatinib-sensitive cell line**. **a** Western blot analysis was employed to detect TrkB protein expression in EC109 cells following lentiviral-mediated *NTRK2* overexpression. **b** IC_50_ values of afatinib in OE-*NC*/OE-*NTRK2* EC109 cells were determined by CCK8 assay. The data are presented as the mean ± s.d. *P* values were calculated using unpaired two-tailed t tests. Western blotting was independently repeated three times with consistent results, and representative images are displayed.


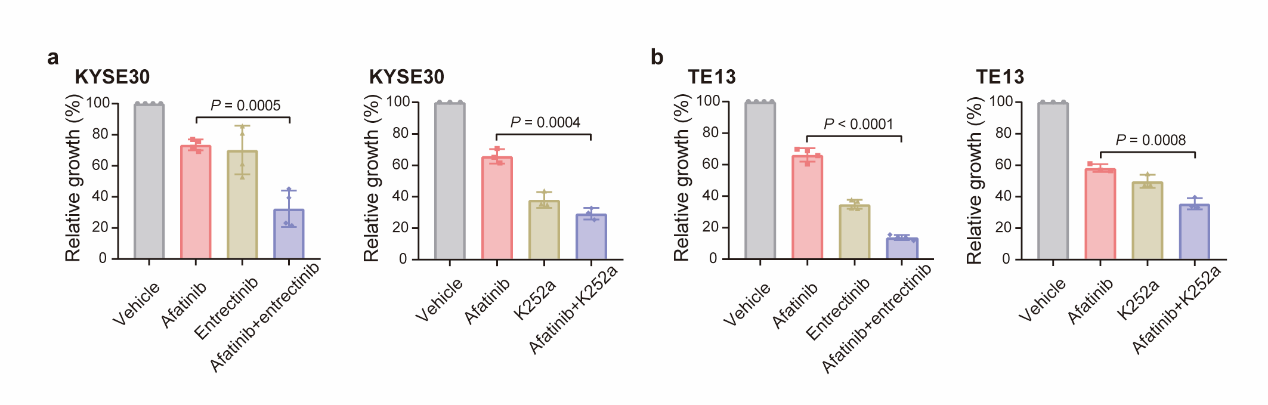


**Supplementary Fig. 6 The impact of combined therapy with pan-Trk inhibitors and afatinib on ESCC cells *in vitro*.** Plots depict the growth of KYSE30 **a** and TE13 **b** treated *in vitro* with either 1μM afatinib, 5μM Entrectinib or 0.5μM K252a alone or in combination. All data are expressed as the percentage of growth relative to that of vehicle-treated control cells. All experiments were repeated at least three times. Data are presented as the mean ± s.d. Student’s *t*-test was used for statistical analysis.
