# Supplementary material for: Clinical efficacy and identification of factors confer resistance to afatinib (tyrosine kinase inhibitor) in EGFR-overexpressing esophageal squamous cell carcinoma
Source: Signal Transduct Target Ther. 2024 Jun 28;9:153. doi: 10.1038/s41392-024-01875-4 (PMC11211462; doi:10.1038/s41392-024-01875-4)
Supplement: Supplementary file 2 — Trial Protocol [file 41392_2024_1875_MOESM2_ESM.docx]

**A Phase II Clinical Study to Evaluate the Efficacy and Safety of Afatinib for Pretreated Patients with Advanced Esophageal Squamous Cell Carcinoma with EGFR Overexpression/Amplification**

**Trial Protocol**

| **Clinical Trial Site** | **Peking University Cancer Hospital and Institute** |
| --- | --- |
| **Clinical Trial Director** | **Shen, Lin, Prof.** |
| **Protocol Version No.** | **Version 3.0** |
| **Protocol Version Date** | **May 20, 2019** |

Table of Contents

[Synopsis of the Study Protocol 3](#_Toc35416583)

[I. Background of the Study 6](#_Toc35416584)

[II. Study Objective 8](#_Toc35416585)

[III. Overall Design of the Study 8](#_Toc35416586)

[IV. Case Selection 8](#_Toc35416587)

[(I) Inclusion Criteria 8](#_Toc35416588)

[(II) Exclusion Criteria 9](#_Toc35416589)

[(III) Removal Criteria 10](#_Toc35416590)

[(IV) Termination Criteria 11](#_Toc35416591)

[V. Clinical Study Assessments 11](#_Toc35416592)

[(I) Screening/Baseline Measurements 11](#_Toc35416593)

[(II) Assessments During Study Treatment 12](#_Toc35416594)

[VI. Collection and Handling of Clinical Study-related Specimens 13](#_Toc35416595)

[VII. Investigational Drug and Other Drugs Used in the Study 13](#_Toc35416596)

[(I) Name and Strength of Study Medications: 13](#_Toc35416597)

[(II) Dosage Regimen: 16](#_Toc35416598)

[(III) Regimen Adjustment 16](#_Toc35416599)

[(IV) Concomitant Medications 16](#_Toc35416600)

[VIII. Treatment Outcome Measures 17](#_Toc35416601)

[IX. Safety Evaluation 17](#_Toc35416602)

[X. Data Management and Statistical Analysis 29](#_Toc35416603)

[XI. Study Management 30](#_Toc35416604)

[XII. Ethics and Laws and Regulations 32](#_Toc35416605)

[XIII. Study Period 34](#_Toc35416606)

[XIV. Study Personnel 34](#_Toc35416607)

[XV. Annexes 34](#_Toc35416608)

# Synopsis of the Study Protocol

| Study Title | A Phase II Clinical Study to Evaluate the Efficacy and Safety of Afatinib for Pretreated Patients with Advanced Esophageal Squamous Cell Carcinoma with EGFR Overexpression/Amplification |
| --- | --- |
| Study Objective | To explore the efficacy and safety of afatinib as the second-line or beyond therapy for advanced esophageal squamous cell carcinoma (ESCC) with EGFR overexpression/amplification |
| Trial Site | Peking University Cancer Hospital and Institute |
| Trial Design | A non-randomized, open-label, single-arm clinical study. Subjects receive afatinib (40 mg po. Qd) in 4-week cycles until disease progression, occurrence of intolerable toxicity, loss to follow-up or death, or other situations in which the investigator determines the treatment should be discontinued. |
| Number of Subjects | 40 |
| Study Endpoints | Primary study endpoint: Objective response rate (ORR)  Secondary study endpoints: Disease control rate (DCR), safety, progression Free Survival (PFS) and overall survival (OS)  Exploratory endpoint: Quality of life, efficacy markers |
| Study Duration | First Patient In Time (FPI): 05/2019  Last Patient In Time (LPI): 05/2020 |
| Inclusion Criteria | 1. Voluntarily participating and signing the Informed Consent Form; 2. Age: 18 to 70 years of age; 3. Male or female; 4. Pathologically confirmed ESCC; 5. Pathologically confirmed immunohistochemical EGFR (3+) or EGFR-FISH amplification or EGFR amplification by NGS-testing (see Annex 4 for specific testing standards) 6. Radiographically confirmed unresectable advanced ESCC; 7. Failure of the first-line or beyond therapy; 8. At least one measurable lesion (per RECIST 1.1 criteria), with the imaging diagnosis ≤ 21 days apart from the enrollment time; 9. Life expectancy of ≥ 3 months; 10. General performance status (ECOG) score of 0-2; 11. Sufficient bone marrow hematopoiesis (within 7 days): hemoglobin ≥ 9 g/dL, white blood cell ≥ 3.0 × 10^9^/L, neutrophil ≥ 1.5 × 10^9^/L, platelet ≥ 100 × 10^9^/L; normal hepatic and renal functions (within 14 days): TBIL ≤ 1.5 × upper limit of normal (ULN); ALT and AST ≤ 2.5 × ULN, or ≤ 5 × ULN in the presence of liver metastasis; creatinine ≤ 1.5 × ULN; 12. Cardiac, pulmonary, renal and hepatic functions are basically normal; |
| Exclusion Criteria | 1. Patients who are currently receiving other effective treatment regimens; 2. Participation in other clinical trials within 4 weeks prior to enrollment; 3. Inability to cooperate in oral administration of afatinib; 4. No measurable tumor lesion, for example, body cavity fluid or diffuse organ infiltration; 5. Prior radiotherapy on the measurable lesion; 6. Coexistence of other primary malignancies other than esophageal cancer, excluding cured basal cell carcinoma of skin and cervical carcinoma in situ; 7. Clinically significant cardiovascular diseases such as heart failure (NYHA class III-IV), uncontrolled coronary heart disease, cardiomyopathy, arrhythmia, uncontrolled hypertension, or history of myocardial infarction within the past 1 year; 8. Nervous or mental disorders affecting the cognitive ability, including central nervous system metastasis; 9. Severe active clinical infection (at grade >2 per NCI-CTCAE Version 4.0) including active tuberculosis within 14 days prior to enrollment; 10. Known or self-complained HIV infection or active hepatitis B or hepatitis C; 11. Uncontrolled systemic diseases such as poorly uncontrolled diabetes mellitus; 12. History of interstitial lung disease such as interstitial pneumonia and pulmonary fibrosis, or chest X-ray/CT evidence of interstitial lung disease at baseline; 13. Keratitis, ulcerative keratitis or severe xerophthalmia; 14. Known hypersensitivity or allergic reaction to any component of the investigational drug; 15. Pregnancy (confirmed by serum β-chorionic gonadotrophin test) or lactation; |
| Treatment Regimen | Afatinib (40 mg Po Qd) therapy in 4-week cycles, until disease progression, occurrence of intolerable toxicity, loss to follow-up or death, or other situations in which the investigator determines the treatment should be discontinued.  Dose adjustment: If a patient cannot tolerate the treatment due to adverse drug reaction, afatinib will be paused until recovering to grade 1 or less, with dose reduction by 10 mg decrements to a minimum dose of 20 mg. |
| Outcome Measures | 1. Efficacy evaluation according to the Response Evaluation Criteria in Solid Tumors (RECIST) 1.1, including CR, PR, SD, PD. ORR (CR+PR), DCR (CR+PR+SD) 2. Efficacy evaluation timepoints: Baseline investigations, specifically, spiral CT or MRI examination (which should remain consistent in subsequent efficacy evaluations), will be done within 3 weeks before treatment, and efficacy evaluation will be performed once at Weeks 4, 8 post-dose, respectively and thereafter once every 8 weeks. In case of disease progression, intolerable toxicity or patient-requested withdrawal, the patient will be allowed to withdraw from the clinical trial. 3. The time window of efficacy evaluation is +/-1 week. |
| Safety and Quality of Life Evaluation | 1. The safety of the investigational drug will be evaluated by recording, reporting and analyzing subjects’ adverse events, vital signs, laboratory tests and other physical examinations. 2. Adverse events will be evaluated according to National Cancer Institute-Common Terminology Criteria for Adverse Events (NCI-CTCAE) Version 4.0 (published on June 14, 2010). 3. The quality of life will be evaluated using EORTC quality of life questionnaires QLQ-C30, QLQ-OES18. |
| Statistical Analysis | All data, including demographics, baseline, various efficacy evaluation indicators and all safety data, etc., will be analyzed by R 3.6.1. |
| Sample Size Calculation | Simon 2-stage design  Power = 0.8; α = 0.05  P_0_ = 0.10, P_1_ = 0.25  Stage 1: N_1_ = 22, R_1_ = 2  Stage 2: N_total_ = 40, R_2_ = 8 |

**A Phase II Clinical Study to Evaluate the Efficacy and Safety of Afatinib for Pretreated Patients with Advanced Esophageal Squamous Cell Carcinoma with EGFR Overexpression/Amplification**

# I. Background of the Study

The morbidity and mortality of esophageal cancer rank 12^th^ and 7^th^, respectively in the global scale. In China, the morbidity and mortality of esophageal cancer rank 6^th^ and 4^th^, respectively, with both the diagnosed cases and death cases reaching 50% of the total in the world, and more than 90% of esophageal cancer are squamous cell carcinoma. The prognosis of esophageal cancer is extremely poor, 70% of patients are at advanced stage of the disease when diagnosed, and their 5-year survival rate is 20% ^[1]^. Traditional cytotoxic chemotherapies for advanced esophageal squamous cell carcinoma (ESCC) is platinum-based. In our center, the combination of paclitaxel and cisplatin as first-line chemotherapy had an objective response rate of 48.6%, a median progression-free survival (mPFS) of 7 months, and a median overall survival (mOS) of 13 months ^[2]^. Esophageal cancer patients who have tumor progression within 6 months after first-line platinum-based chemotherapy cannot receive platinum-based treatment in the second-line therapy as they are considered as resistant to platinum. For such platinum-resistant patients, second-line chemotherapy has a relatively low response rate. Burkart *et al.* reported that, the ORR of irinotecan in cisplatin-refractory advanced esophageal cancer was less than 15% ^[3]^. According to unpublished retrospective data from Gastrointestinal Department of Beijing Cancer Hospital, in the second-line therapy of esophageal cancer using irinotecan, fluorouracil or gemcitabine, etc., the ORR was only 12.8%, mPFS 3.5 months, and mOS 9.3 months. Other than cytotoxic chemotherapy, targeted therapy and immunotherapy are also the hot spots of current research; however, targeted therapy of ESCC has not yet been supported with any positive results from phase III clinical studies and the onset of effect in immunotherapy takes a relatively long time, suggesting neither of them is applicable to patients who develop primary drug resistance rapidly.

Gefitinib, erlotinib and afatinib are small-molecule EGFR tyrosine kinase inhibitors (EGFR-TKIs) that competitively bind to EGFR via endogenous ligands, inhibits the activation of tyrosine kinase and subsequently blocks EGFR signaling pathway to inhibit the proliferation and metastasis of tumor cells and promote their apoptosis. NCCN guidelines recommend the use of the EGFR-TKIs mentioned above as the first-line therapy for EGFR-mutant non-small cell lung cancer ^[4]^. Among them, afatinib is an irreversible dual inhibitor targeting EGFR and human epidermal growth factor receptor 2 (HER2) tyrosine kinases. It has been demonstrated in LUX-Lung 3 and LUX-Lung 6 studies that, compared to chemotherapy, afatinib significantly prolonged the progression-free survival of patients with EGFR-mutant non-small cell lung cancer and significantly reduced the risk of death ^[5, 6]^. In terms of safety, the study results of LUX-Lung 3, LUX-Lung 6 and LUX-Lung 7 showed that, adverse reaction related therapy discontinuation occurred in 8%, 6.3%, and 6.3% of patients, respectively ^[5-7]^. In addition, after dose adjustment of afatinib, adverse reactions were reduced with no influence on the efficacy.

In ESCC, the reported epidermal growth factor receptor (EGFR) overexpression rate is 30-50% and the EGFR amplification rate about 7-13%. Studies have shown that, EGFR overexpression is a contributor to the poor prognosis of esophageal cancer ^[8-11]^. In ESCC, patients with high EGFR expression or those with EGFR amplification can benefit from gefitinib ^[12, 13]^. In EGFR-FISH positive esophageal cancer, overall survival was improved with gefitinib compared with placebo (HR 0.59; 95%CI 0.35-1.00, P = 0.05), and patients with EGFR amplification gained greater benefit from gefitinib (HR 0.21; 95%CI 0.07-0.64, P = 0.006). In preclinical study, afatinib achieved the best efficacy in PDX models with EGFR overexpression, especially EGFR amplification ^[14]^. So far, there have been no clinical study reports on the efficacy and safety of afatinib in treating ESCC.

Based on the study background above, this study explores the efficacy and safety of afatinib for pretreated patients with advanced ESCC with EGFR overexpression/amplification and provides advanced ESCC patients with more evidence-based medical proof through prospective exploration with a small sample size.

# II. Study Objective

To explore the efficacy and safety of afatinib for pretreated patients with advanced ESCC with EGFR overexpression/amplification.

# III. Overall Design of the Study

This study is a non-randomized, open-label, single-arm clinical study. Subjects receive afatinib (40 mg po. Qd) in 4-week cycles until disease progression, occurrence of intolerable toxicity, loss to follow-up or death, or other situations in which the investigator determines the treatment should be discontinued.

# IV. Case Selection

## (I) Inclusion Criteria

- - - 1. Voluntarily participating and signing the Informed Consent Form (ICF);
      2. Age: 18 to 70 years of age;
      3. Male or female;
      4. Pathologically confirmed ESCC;
      5. Pathologically confirmed immunohistochemical EGFR (3+) or EGFR-FISH amplification or NGS-testing EGFR amplification
      6. Radiographically confirmed unresectable advanced ESCC;
      7. Failure of the first-line or beyond therapy;
      8. At least one measurable lesion (per RECIST 1.1 criteria) or evaluable but non-measurable lesion, with the imaging diagnosis ≤ 21 days apart from the enrollment time;
      9. Life expectancy of ≥ 3 months;
      10. General performance status (ECOG) score of 0-2;
      11. Sufficient bone marrow hematopoiesis (within 7 days): hemoglobin ≥ 9 g/dL, white blood cell ≥ 3.0 × 10^9^/L, neutrophil ≥ 1.5 × 10^9^/L, platelet ≥ 100 × 10^9^/L; normal hepatic and renal functions (within 14 days): TBIL ≤ 1.5 × upper limit of normal (ULN); ALT and AST ≤ 2.5 × ULN, or ≤ 5 × ULN in the presence of liver metastasis; creatinine ≤ 1.5 × ULN;
      12. Cardiac, pulmonary, renal and hepatic functions are basically normal;

## (II) Exclusion Criteria

1. Patients who are currently receiving other effective treatment regimens;
2. Participation in other clinical trials within 4 weeks prior to enrollment;
3. Inability to cooperate in oral administration of afatinib;
4. No measurable tumor lesion, for example, body cavity fluid or diffuse organ infiltration;
5. Prior radiotherapy on the measurable lesion;
6. Prior use of anti-EGFR targeted therapy;
7. Coexistence of other primary malignancies other than esophageal cancer, excluding cured basal cell carcinoma of skin and cervical carcinoma in situ;
8. Clinically significant cardiovascular diseases such as heart failure (NYHA class III-IV), uncontrolled coronary heart disease, cardiomyopathy, arrhythmia, uncontrolled hypertension, or history of myocardial infarction within the past 1 year;
9. Nervous or mental disorders affecting the cognitive ability, including central nervous system metastasis;
10. Severe active clinical infection (at grade >2 per NCI-CTCAE Version 4.0) including active tuberculosis within 14 days prior to enrollment;
11. Known or self-complained HIV infection or active hepatitis B or hepatitis C;
12. Uncontrolled systemic diseases such as poorly uncontrolled diabetes mellitus;
13. History of interstitial lung disease such as interstitial pneumonia and pulmonary fibrosis, or chest X-ray/CT evidence of interstitial lung disease at baseline;
14. Keratitis, ulcerative keratitis or severe xerophthalmia;
15. Known hypersensitivity or allergic reaction to any component of the investigational drug;
16. Pregnancy (confirmed by serum β-chorionic gonadotrophin test) or lactation;

## (III) Removal Criteria

1. Non-compliance with inclusion criteria;
2. Patients who violate the requirements of the study protocol, fail to take medicines as required and fail to complete the plan;
3. Poor quality of data recording, and incomplete and inaccurate data.
4. Use of other antitumor drug therapy during the clinical study period.

## (IV) Termination Criteria

Patients should terminate the study treatment in any of the following cases:

1. Disease progression determined by investigator;
2. Occurrence of intolerable toxicity;
3. Treatment is delayed 14 days or more due to adverse reaction or other reasons;
4. Patient’s compliance is poor;
5. The investigator believes that patient is not suitable for continuing such treatment or the patient cannot benefit from the treatment;
6. Subject requests withdrawal.

Once a subject withdraws from the study, the investigator must record the reason for withdrawal in his/her case report form or medical record. All subjects who withdraw from the study due to adverse event or clinical laboratory abnormality must be followed up until recovered or stable, with their subsequent outcome recorded.

# V. Clinical Study Assessments

## (I) Screening/Baseline Measurements

| Assessment Items | | Time Prior to First Dose |
| --- | --- | --- |
| 1. ICF | Signing | Prior to first dose |
| 2. Medical history and physical examination | Medical history: Diagnosis of ESCC, concomitant medications, history of hypersensitivity to the investigational drug, and general past medical history, including concomitant diseases.  Physical examination: Body height, body weight, ECOG performance status score (see Annex 1 for details), current symptoms, nervous system examination. | 14 days |
| 3. Routine blood test | Hemoglobin, platelet, white blood cell, neutrophil | 7 days |
| 4. Biochemistry | Serum biochemistry (ALT, AST, AKP, BIL, BUN, Crea, ALB, TG, TCHO, CRP, electrolytes) | 14 days |
| 5. Routine urine test | Urine PH, urine protein, urine red blood cell, urine white blood cell | 14 days |
| 6. Imaging* | CT or MRI | 21 days |
| 7. Electrocardiogram | 12-lead ECG | 21 days |
| 8. Pathology | Endoscopic biopsy and pathology | No limit |
| 9.Quality of life evaluation | EORTC QLQ-C30 and EORTC QLQ-OES18 | 3 days |
| * To evaluate the efficacy, pre-treatment and post-treatment CT or MRI scans should be performed by the same methods for comparison. | | |

## (II) Assessments During Study Treatment

| **Assessment Items** | | **Time** |
| --- | --- | --- |
| 1. Physical examination | Body height, body weight, ECOG performance status score, concomitant therapies and medications | Once every 4 weeks |
| 2. Routine blood test* | Hemoglobin, platelet, white blood cell, neutrophil | Once weekly |
| 3. Biochemistry* | Serum biochemistry (ALT, AST, AKP, BIL, BUN, Cr), electrolytes (K^+^, Na^+^, Cl^-^, Ca^2+^, Mg^2+^) | Once every 2 weeks |
| 4. Imaging** | ·CT or MRI scan  ·Other clinically indicated examinations | Efficacy evaluation will be performed once at Weeks 4, 8 post-dose, respectively and thereafter once every 8 weeks |
| 5. Adverse event and quality of life evaluation*** | See the annexes for specific items and grading | Adverse events will be observed and recorded throughout the trial; quality of life will be evaluated once every 4 weeks |
| 6. Quality of life evaluation | EORTC QLQ-C30 and QLQ-OES18 scores | Once every 3 weeks |
| *Since Week 1 of treatment, the time window of the test is ±3 days.  **For patients whose efficacy is evaluated as CR or PR, efficacy evaluation should be performed again in 4 weeks for confirmation; efficacy evaluation will be completed within 1 week following the examination of tumor outcome measures.  ***The observation period collecting adverse events is from the administration of first dose to 30 days after the last dose. Serious adverse events will be collected and recorded according to the protocol. | | |

# VI. Collection and Handling of Clinical Study-related Specimens

During this study, subject’s related samples for routine blood test, serum biochemistry, blood coagulation and routine urine test, etc. will be collected by the investigator and professional personnel in a scientific and standard manner and preserved in the Laboratory Department of Beijing Cancer Hospital. The above test samples will be destructed by the site within 14 days after the test results are confirmed.

# VII. Investigational Drug in the Study

## (I) Name and Strength of Study Medications:

**1. Afatinib Dimaleate (Giotrif)**

**1.1 General information:**

a) Dosage form: Tablets, 40 mg/tablet;

b) Dosage and administration: 40 mg Qd; in case of intolerable adverse drug reaction, the dose will be adjusted to 30 mg Qd or 20 mg Qd. If the patient cannot tolerate 20 mg/day, consider permanent discontinuation of afatinib.

c) Storage condition: Store in a tightly closed container.

**1.2 Instructions for use:**

a) Afatinib should not be taken with meal. Take afatinib at least 3 hours after or 1 hour before a meal and swallow the tablet as whole with water;

b) Dose reduction can decrease the incidence of common adverse reactions;

c) Adequate contraception should be taken during the treatment period and until at least 2 weeks after the last dose;

**1.3 Adverse reactions:**

a) Gastrointestinal reactions: Diarrhea has resulted in drug withdrawal in about 2% of patients. Diarrhea usually occur within the first 2 weeks of treatment and may result in dehydration with or without renal impairment. Therefore, anti-diarrheal medicinal products (e.g., Loperamide) should be used at first signs of diarrhea. Patients with severe diarrhea (grade 2 or 3 diarrhea persisting more than 48 hours) may require interruption and dose reduction or discontinuation of therapy with afatinib.

b) Skin related adverse events: Rash/ache has resulted in drug withdrawal in about 2% of patients and generally manifests as a mild or moderate erythematous and acneiform rash, which may occur or worsen in areas exposed to sun. For patients who are exposed to sun, protective clothing, and/or use of sun screen is advisable. Early intervention (such as emollients, antibiotics) of dermatologic reactions is recommended. For patients with severe bullous, blistering or exfoliative sin conditions, discontinue afatinib therapy permanently and refer to a specialist with expertise in managing these dermatologic effects.

c) Interstitial lung disease: Interstitial lung disease (ILD) or ILD-like adverse reactions (e.g., lung infiltration, pneumonitis, acute respiratory distress syndrome or allergic alveolitis) are reported in 1.6% of patients treated with afatinib, and 0.4% of them die. Interrupt afatinib therapy in all patients with acute onset and/or unexplained worsening of pulmonary symptoms (dyspnea, cough, fever). If ILD is diagnosed, discontinue afatinib permanently and refer to a respiratory specialist in managing the condition.

d) Hepatic function: Hepatic failure has been reported during treatment with afatinib in 1% of patients. Periodic liver function testing is recommended.

e) Keratitis: Symptoms such as acute or worsening eye inflammation, lacrimation, light sensitivity, blurred vision, eye pain and/or red eye should be referred promptly to an ophthalmology specialist. If a diagnosis of ulcerative keratitis is confirmed, treatment should be interrupted.

**1.4 Contraindications:**

Contraindicated in patients with known hypersensitivity to afatinib or to any of the excipients.

**1.5 Drug interactions:**

a) Concomitant taking of strong P-glycoprotein (p-gp) inhibitors (e.g., cyclosporine A, ketoconazole, itraconazole, erythromycin, verapamil, quinidine, tacrolimus, amiodarone, etc.) with afatinib can increase exposure to afatinib;

b) Concomitant taking of strong p-gp inducers (e.g., rifampicin, carbamazepine, phenytoin, phenobarbital, or St. John’s wort) with afatinib can decrease exposure to afatinib;

**1.6 Food effect on afatinib:**

a) Co-administration of a high-fat meal with afatinib can result in a significant decrease of exposure to afatinib

## (II) Dosage Regimen:

Afatinib (40 mg Po Qd) therapy, 4-week cycles, until disease progression, occurrence of intolerable toxicity, loss to follow-up or death, or other situations in which the investigator determines the treatment should be discontinued.

## (III) Regimen Adjustment

Dose adjustment of afatinib

| CTCAE drug-related adverse events | Recommended dose of afatinib |
| --- | --- |
| Grade 1 or 2 | Do not interrupt the treatment or adjust the dose |
| Grade 2 (prolonged, for example, diarrhea > 48 hour and/or rash > 7 days, or intolerable) or ≥ Grade 3 | Interrupt the treatment until the event is resolved to grade 0/1, and then restore the treatment with the dose reduced by 10 mg. If the patient cannot tolerate 20 mg/day, consider permanent discontinuation of afatinib. |

If a patient’s treatment is delayed due to adverse reaction but there is no explicit evidence of tumor progression, the clinical study can be continued no matter how long the treatment is delayed.

## (IV) Concomitant Medications

**1. Permitted Medications**

For the treatment of pain, infections and other tumor complications and/or study treatment-related toxicities during the study, sedatives, antibiotics, analgesics, antihistamines, steroids, granulocyte colony-stimulating factor and transfusion of red blood cells, platelets or fresh frozen plasma may be administered.

**2. Prohibited Medications**

During the study, subjects are not allowed to use Chinese medicines with an antitumor effect indicated in the package insert; and not allowed to receive concomitant, long-term systemic immunotherapy, chemotherapy, radiotherapy (except palliative radiotherapy), hormonal therapy for treatment of tumors (however, glucocorticoids for antiemetic purpose and progestogen for tumor cachexia are exceptions), or other investigational drugs.

# Treatment Outcome Measures

1. Efficacy will be evaluated according to the Response Evaluation Criteria in Solid Tumors (RECIST) 1.1, including CR, PR, SD, PD. ORR (CR+PR), DCR (CR+PR+SD). Efficacy evaluation timepoints: Baseline investigations, specifically, spiral CT or MRI examination (which should remain consistent in subsequent efficacy evaluations), will be done within 3 weeks before treatment, and efficacy evaluation will be performed once at Weeks 4, 8 post-dose, respectively and thereafter once every 8 weeks. In case of disease progression, intolerable toxicity or patient-requested withdrawal, the patient will be allowed to withdraw from the clinical trial.
2. Adverse events will be evaluated using the National Cancer Institute-Common Terminology Criteria for Adverse Events (NCI-CTCAE) Version 4.0 (published on June 14, 2010).
3. Pre-treatment Formalin-fixed paraffin-embedded (FFPE) tumor tissue samples will be collected from each patient to explore efficacy biomarkers.

# IX. Safety Evaluation

The safety endpoints include:

- Incidence of all types of AE
- Mortality and causes of death
- Safety laboratory tests, and where applicable, grading per NCI-CTCAE (Version 4.0)
- Vital signs

Safety measures

Symptoms: General symptoms (fatigue, asthenia, perspiration, flushing, fever, lump “sensation”), nervous system symptoms (dizziness, drowsiness, headache, depression, epilepsy, confusion), cardiovascular system symptoms (tachycardia, bradycardia, thromboembolism chest pain), respiratory system symptoms (cough, shortness of breath, dyspnea, asthma), digestive system symptoms [anorexia, nausea, vomiting, abdominal pain, diarrhea, gastrointestinal perforation), hepatotoxicity (hepatic necrosis, chronic hepatitis, hyperbilirubinemia), lipase and amylase increased, pricking pain of tongue tip], hematological symptoms [anemia, hemorrhage, hematological toxicity (thrombocytopenia, neutropenia)], thyroid function abnormalities (hyperthyroidism/hypothyroidism), skin mucosal symptoms (rash, ulceration, pruritus, congestion, etc.), alopecia, injection site symptoms (red swelling, thermalgia and phlebitis), infusion reactions and other symptoms. Special attention should be paid to the sequential relationship between these symptoms and the investigational drug, and their changing process.

Vital signs and physical examination: Mainly include body height, body weight, body surface area, ECOG score, body temperature, blood pressure, heart rate, and respiratory rate.

Laboratory tests: Mainly include routine blood test (Hb, WBC, ANC, PLT), routine urine test (urine PH, urine protein, urine red blood cell, urine white blood cell), hepatic function (ALT, AST, TBIL), renal function (BUN, Cr), electrolytes (K^+^, Na^+^, Cl^-^, Ca^2+^, Mg^2+^), etc.

Auxiliary examinations: Electrocardiogram, X-ray, ultrasound or CT/MRI, etc.

**9.1 Adverse Event (AE)**

The investigator will be responsible for detecting, recording and reporting various types of events that fit the definition of AE or SAE.

**9.1.1 Definition of AE**

An AE is any adverse medical occurrence in a clinical trial subject administered a pharmaceutical product. In this trial, any adverse medical events that occur since the initiation of investigational drug treatment till 30 days after end of treatment will be deemed as AE, no matter they have a causal relationship with the investigational drug or not.

Note: An AE can therefore be any unfavorable and unintended sign (including a clinically significant abnormal laboratory finding), symptom, or disease temporally associated with the use of the investigational drug (newly emergent disease, or worsening of primary disease); for commercially available pharmaceutical products, may also include absence of expected efficacy after administration (e.g., lack of efficacy), abuse and misuse

Examples of AE include:

- Deterioration of chronic sickness or interrupted onset of original symptoms, including increase in the frequency and/or intensity of symptom onset;
- New symptoms detected or diagnosed after administration of the investigational drug, even if such symptoms may have existed before the initiation of the study;
- Signs, symptoms or clinical sequelae suspected to be caused by interactions;
- Signs, symptoms or clinical sequelae caused by the overdose of investigational drug or concomitant medications (essentially, overdose should not be reported as AE/SAE);
- In case of patient death, the cause of death is AE, and the death is the outcome of the AE.

Examples of AE do not include:

- Medical or surgical procedures (e.g.: endoscopy, appendectomy); conditions that lead to such procedures are AE, but surgical procedures themselves are not AE;
- Circumstances in which no adverse medical event occurs (social and/or allowable to hospitals);
- Premature daily fluctuation of pre-existing disease, or no deterioration of symptoms existing or detected at the beginning of the study;
- On-study sickness/disease, or anticipated progression, signs or symptoms of on-study sickness/disease, unless worse than the subject’s anticipated condition;
- Principally, “lack of efficacy” or “absence of intended pharmacological action” should not be reported as AE/SAE. However, signs, symptoms and/or clinical sequelae caused by lack of efficacy, if meeting the definition of AE or SAE, will also be reported as AE/SAE.

**9.1.2 Definition of Severe Adverse Event (SAE)**

An SAE refers to any adverse medical event that at any dose:

1. results in death
2. is life-threatening

Note: The term “life-threatening” in the definition of “serious” refers to an AE in which the patient was at risk of death at the time of the event. However, it does not refer to an AE which hypothetically might have caused death if it were more severe.

1. requires inpatient hospitalization or causes prolongation of existing hospitalization

- Note: Generally, hospitalization refers to subject’s stay in the hospital or emergency ward (at least overnight stay) for observation or treatment, while such observation or treatment should not be performed in the clinic or outpatient section. Complications which occur during the hospitalization period are AE. If a complication prolongs the existing hospitalization or complies with any other criteria of SAE, the event is serious. Whenever there is any doubt about “hospitalization” or necessity of hospitalization, AE should be considered serious.
- The elective treatment of underlying disease with no aggravation from baseline should not be considered as AE

1. Results in persistent or significant disability/incapacity

Note: The term “disability” refers to significant loss in personal activities of daily living. This definition does not include symptoms of less medical significance such as headache, nausea, vomiting, diarrhea, influenza or accidental trauma (ankle sprain), which will interfere with daily living, but will not cause significant loss of function.

1. is a congenital anomaly/birth defect
2. Medical and scientific judgment should be exercised in deciding whether reporting is appropriate in other situations. For example:

- Important medical events that may not be immediately life-threatening or result in death or hospitalization but may jeopardize the patient or may require intervention to prevent one of the other outcomes listed in the definition above should also usually be considered SAE. Examples of such events include malignancies, allergic bronchospasm that requires emergency treatment in an emergency room or at home, blood dyscrasias or convulsions that do not result in hospitalization, or development of drug dependency or drug abuse. An AE which is considered serious in the health expert’s report (e.g.: be considered to result in functional disorder even not complying with the definition of SAE above) should also be reported as SAE.
- For the purpose of reporting, pathogen transmission caused by the route of study administration should also be considered SAE, and all such reaction should be subject to expedited reporting.

**9.1.3 Disease-related Events or Results Not Considered as SAE**

If an event is a part of the natural course of the disease under study (i.e., disease progression), it should not be reported as SAE. The progression of tumor lesion should be recorded in the clinical evaluation part of the eCRF. Death due to disease progression should be recorded in the “Death Record” page of the eCRF and should not be reported as SAE. However, if a subject’s progression of underlying disease exceeds normal expectations, or if the investigator considers there is a causal relationship between the use of investigational drug treatment or study protocol design/procedure and disease progression, the progression must be reported as SAE. Any newly emergent primary tumor must be reported as SAE.

**9.1.4 Abnormalities of Laboratory Tests and Other Safety Assessments Reported as AEs and SAEs**

Any abnormal findings of laboratory tests (hematology, clinical biochemistry or urine test) or other safety assessments (e.g., electrogram, imaging, measurement of vital signs), including results with abnormal changes from baseline, and examination results which are considered clinically significant by the investigator based on medical and scientific judgment, should be reported as AE or SAE.

However, any important clinical safety assessments related to the underlying disease will not be reported as AE or SAE, unless the investigator considers the assessment results are worse than expectations.

**9.1.5 Time Period and Frequency for Collection of AE and SAE Information**

In this study, the information of all AEs that occur within the period from the administration of the first dose of investigational drug till 30 days after the last dose, regardless of their relationship with the investigational drug, will be collected and recorded in the eCRF.

SAEs related to participation in the study (including prejudicial examinations in the study process or for screening), whether or not related to the investigational drug, will be collected and recorded since the subject’s consent on participation in this study till 30 days after the last dose of the investigational drug.

If considered possibly related to the investigational drug, even SAEs which occur 30 days after the last dose should be reported.

All AEs/SAEs, once occur, will be followed up in a strict manner at each specified visit of the study, investigator’s each contact with the subject, and visits after end of study. The investigator will collect AE/SAE information by asking the standard questions below during the study period and 30-day follow-up period and at each visit:

How are you feeling?

Do you have any (other) medical problems since the last visit?

Do you take any new medicines since the last follow-up/assessment?

**9.1.6 Recording of AE and SAE**

When an AE/SAE occurs, the investigator is responsible for reviewing all documentations related to the event (e.g., progress notes, laboratory tests and diagnostic reports). And then record all AE/SAE related information in the eCRF. The investigator is not allowed to submit the subject’s medical record copy as AE/SAE eCRF report form. However, when the medical record copy of some cases is required, this may happen, in which case, all the subject identifications on the medical record copy should be masked before submission.

The investigator should endeavor to make a definitive diagnosis based on the signs, symptoms and/or other clinical information. The diagnosis, instead of single sign or symptom, should be recorded as AE/SAE.

Any AEs or SAEs occurring during the study period must be recorded in the subject’s case file and corresponding eCRF. Each AE or SAE must be separately recorded. Death due to disease progression should be recorded in the “Death Record” page of the eCRF and should not be recorded as SAE.

If abnormal laboratory findings or other abnormal assessments of clinical significance meet the definition of AE or SAE, the AE eCRF page or SAE eCRF page should be completed as required. A definitive diagnosis (if known), instead of abnormal finding, should be recorded.

**9.1.7 Criteria for Assessing the Severity of AE**

The investigator will assess AEs by referring to National Cancer Institute-Common Terminology Criteria for Adverse Events (NCI-CTCAE) Version 4.0 (Version Date: June 14, 2010, see Annex 4). NCI-CTCAE V4.0 adopts descriptive terminology for reporting AEs. The investigator is required to grade the severity of each AE.

If the severity/intensity of a certain kind of AE is not clarified in this guideline, the investigator can make assessments according to the general definitions of Grades 1 to 5 and based on his/her best medical judgment.

AEs are generally graded as:

Grade 1: mild

Grade 2: moderate

Grade 3: severe

Grade 4: life-threatening or resulting in disability

Grade 5: death related to AE*

If the severity/intensity of a certain AE reaches Grade 4, the investigator must report the event as SAE according to the procedures defined in Section 9.1.11 (see the definition in Section 9.1.2). However, Grade 4 laboratory abnormalities such as anemia or neutropenia will be considered SAE only when they meet the criteria of severity described below.

* Note: It is generally considered that death (defined as Grade 5 per NCT-CTCAE Version 4.0) is the outcome of an event and should be recorded as described below.

In case of death, the leading cause of death (the main cause of death) will be recorded and reported as SAE. “Life-threatening” will be recorded as the outcome of the AE; death should not be recorded as an independent event. Only when the cause of death remains uncertain (e.g.: sudden death, unexplained death) can death itself be reported as SAE.

**9.1.8 Pregnancy**

Pregnancy that occurs in the study period must be recorded in the pregnancy report form and reported. To ensure subject’s safety, pregnancy must be reported to investigator within 2 weeks following its occurrence, and must be followed up to determine the outcome (including early termination of pregnancy) and maternal and infant status. Pregnancy complications and termination of pregnancy for medical reasons must be reported as AE/SAE. Spontaneous abortion must be reported as SAE.

In addition, the investigator must try their best to collect the pregnancy information of a male subject’s female sex partner after study enrollment. Pregnancy information must be reported as described above.

**9.1.9 Judgment on the Correlation between AE and Investigational Drug**

The relationship between the investigational drug and AE or the role of the investigational drug in an AE can be judged according to the following categories and criteria:

1) Not related: Through thorough medical judgment, the AE is caused by other reasons (sickness, environment, etc.);

2) Unlikely related: Through thorough medical judgment, the AE is considered unlikely related to the investigational drug:

- There is no temporal relation between the onset of the AE and the administration of the drug
- The AE may be caused by other factors such as change in the disease course, environment or administration of other therapeutic drugs
- The onset of the AE is unrelated to the known characteristics of the drug
- The AE does not recur or worsen after re-challenge

3) Possibly related: (the first 2 must apply) If through thorough medical judgment, the following conditions are met, the AE is considered possibly related to the investigational drug:

- There is a temporal relation between the onset of the AE and the administration of the drug
- The relationship between the onset of AE and the disease course, environment, toxicity or administration of other therapeutic drugs cannot be excluded
- The AE corresponds to the known characteristics of the drug

4) Related: (the first 3 must apply) If through thorough medical judgment, the following conditions are met, the AE is considered related to the investigational drug:

- There is a temporal relation between the onset of the AE and the administration of the drug
- The AE cannot be explained by the disease course, environment, toxicity or administration of other therapeutic drugs
- After drug withdrawal or dose reduction, the AE disappears or abates, and recurs after re-challenge
- The AE corresponds to the known characteristics of the drug

5) Definitely related: (the first 4 must apply) If through thorough medical judgment, the following conditions are met, the AE is considered definitely related to the investigational drug:

- The AE corresponds to the transient outcome after administration, or drug level is detected in body fluid tissues
- The AE cannot be explained by the subject’s disease course, environment, toxicity or administration of other therapeutic drugs
- After drug withdrawal or dose reduction, the AE disappears or abates, and recurs after re-challenge
- The AE corresponds to the known characteristics of the drug

**9.1.10 Immediately Reported SAEs and Other Events**

Once determining an event meets the definition of event in the study protocol, the investigator must report the event promptly as described in the table below

|  | Initial Report | | Follow-up Report | |
| --- | --- | --- | --- | --- |
| Type of Event | Time Frame | Document | Time Frame | Document |
| All SAEs | 24 hours | “SAE” information collection tool | 24 hours | Updated SAE information collection tool |
| Pregnancy | 2 weeks | Pregnancy report form | 2 weeks | Updated pregnancy form |

The methods for detecting, recording, assessing and following up AEs and SAEs and procedures for completing and submitting SAE report are provided in the SMP. The procedures for the reporting of AEs/AEs occurring after the end of study are also included.

**9.1.11 SAE Reporting Process**

For any SAEs occurring within the reporting period, the investigator must report it immediately (i.e., within a maximum of 24 hours following the awareness of the event) by phone or fax.

After the event (or follow-up information) is reported by phone, it should also be immediately reported to the Ethics Committee of Beijing Cancer Hospital, China Food and Drug administration, and the food and drug administration bureau of where the study site locates (province or city) by fax. For previously reported SAE, if any new information is available, the reporting process and time frame are the same as before (follow-up).

For the name, address, telephone number and fax number of SAE reporting, please refer to the information in the SAE report form. All written reports must be uploaded in the form of SAE report form and completed by the investigator according to the specified instructions for completion.

The investigator/reporter must timely updata follow-up information (e.g.: other information, outcome and final assessment, special records required).

Normally the clinical research associate in charge will make a request for access to follow-up information, but in special cases, the Global Drug Safety Department may contact the investigator directly to confirm, corroborate or discuss a certain important event.

The recording of SAE should specify the symptoms, severity, occurrence time, handling time, action taken, follow-up time and method, and outcome profile.

# X. Data Management and Statistical Analysis

**10.1 Completion and Transfer of Case Report Form**

The Case Report Form (CRF) will be completed by the doctor, and must be completed separately for each case enrolled. After completion, the CRF will be reviewed by the clinical research associate, and then its first copy will be transferred to the data manager for data entry and management work.

**10.2 Data Entry and Modification**

An independent data management unit will be responsible for data entry and management. To guarantee the accuracy of data, double entry and proofread should be performed by two data managers independently. For questions in the CRF, the data manager will fill out a Data Request Queue (DRQ), and send a query to the investigator via the clinical research associate, to which the investigator should answer the questions and reply as soon as possible, and then the data manager will carry out data modification, confirmation and entry according to the investigator’s answer, and if necessary, send a DRQ again.

**10.3 Statistical Analysis of Trial Data**

All the data will be statistically analyzed and reported using R ver. 3.6.1 software. All patients who have received at least one therapeutic dose will be included in the descriptive safety analysis. Safety analysis: predominantly descriptive statistical analysis.

# XI. Study Management

**1. Case Report Form**

The primary purpose is to obtain information required by the study protocol in a complete, accurate, clear and timely manner. The data in the CRF should be consistent with those in the source documents. The CRF must be completed in a complete and legible manner (using black or blue ball pain, complying with the requirements in regulatory documents).

All revisions and corrections must be done and confirmed by the investigator, with the date of revision/correction specified. Mistakes must be retained clearly and not overwritten with corrected data (e.g., using correction fluid). The investigator must indicate the reason for revising important information.

For missing information/annotation in the medical record, their space input in the CRF should be substituted by line-drawing cancellation to avoid unnecessary follow-up investigation.

The CRF is a regulatory document and must be appropriate for submission to the hospital authorities.

**2. Study File and Preservation**

The investigator should have a file concerning the study objective. This file should contain all related documents required for conducting the study. After the end of the study, these documents should be archived according to relevant regulations of the hospital and the country.

**3. Regulations on Data Management and Data Traceability**

The CRF will be completed by the investigator and must be completed separately for each enrolled subject. After completion, the CRF will be reviewed by the clinical research associate, and then transferred to the data manager for double data entry and data management work.

During this period, query forms may be transferred by the clinical research associate to the investigator for data review, the investigator should answer the queries and return the form so soon as possible. It is not allowed to modify locked data documents. The database will be transferred to the statistical analysis personnel, who will then carry out statistical analysis according to the requirements in the statistical plan. The statistical analysis report will be given to the principal investigator of this trial for writing the study report.

The investigator will be trained on relevant laws and regulations and SOP, and all investigators are required to strictly abide by such laws and regulations and SOP, so as to ensure the data are recorded in an authentic, timely, accurate and complete manner and prevent omission and arbitrary modification, data falsification and data fabrication; the clinical research associate should strengthen the verification of various records. Subject’s follow-up medical record is one of the source documents and should be preserved together with the CRF. The laboratory department of each site provides the quality control certification granted by the National Center for Clinical Laboratories, and the laboratory results will be preserved in the computer connected to the test equipment for at least three years for further reference.

**4. Quality Control and Quality Assurance of Clinical Trial**

Both the sponsor and investigator should perform their respective duties, strictly follow the clinical trial protocol, and adopt standard operating procedures to guarantee the implementation of the quality control and quality insurance system of the clinical trial.

All related observations and findings in the clinical trial should be verified and each stage of data processing must be performed with quality control to ensure the completeness, accuracy, authenticity and reliability of data.

This study is subject to systemic inspection by drug regulatory agencies and sponsor’s authorized inspector on the related activities and documents of this clinical trial to evaluate if this trial is conducted in accordance with the trial protocol, standard operating procedures and relevant regulatory requirements and if the trial data are recorded in a timely, authentic, accurate and complete manner. The inspection should be executed by personnel not directly involved in this clinical trial.

This study is subject to the inspection by drug regulatory agencies on the respective duties of the investigator and sponsor in implementing this trial and fulfillment of such duties.

**5. Other Regulations**

The CRF will be completed by the investigator and must be completed separately for each case enrolled. After completion, the CRF will be reviewed by the clinical research associate, and then transferred to the data manager for data entry and management work.

# XII. Ethics and Laws and Regulations

**1. Investigator’s Responsibilities**

The investigator should be responsible for ensuring that the clinical study is conducted in accordance with the study protocol and that the ethical principles in the Declaration of Helsinki (World Medical Association Declaration of Helsinki, current revised version) and Good Clinical Practice (GCP) with appropriate modifications in line with China's actual conditions. Subject-related materials and specimens will be preserved in Beijing Cancer Hospital, scientific and standard information management and specimen collection will be carried out by the investigator and professional personnel, and only the investigator, Ethics Committee and drug regulatory agencies will be given access to related medical records. After the end of the study, related specimens will be disposed of according to the hospital’s routine process. Any public reports concerning the results of this study will not disclose subject’s personal identity. We will make every effort to protect subject’s personal medical information privacy to the extent permitted by law. The above documents must be expounded in the subject’s ICF, which is the essential prerequisite for clinical study enrollment.

**2. Subject Information**

Signing the written ICF is the essential prerequisite for a patient to be enrolled. Before the signature, the investigator must provide subjects with sufficient information. If in compliance with the hospital’s regulations, the investigator may designate a person to provide subjects with such information. Besides from the provision of written information, the investigator or designee should also verbally inform the patients. Attention should be paid to the selection of wording, so that subjects can fully and easily understand the information.

Whenever any new important information involving patient’s informed consent is found, the subject information form should be modified accordingly.

**3. ICF**

Before any study-related behavior is conducted, there must be a written consent form on patient’s voluntary participation in the clinical trial. The ICF should be signed by the patient and the investigator or his/her designee in person, with the date of signature indicated.

The investigator should confirm in the CRF again whether a consent form is signed. The signed and dated ICF should be preserved at the study site and archived safely by the investigator for supervision, review and inspection at any time.

An original copy of the ICF should be given to patients before the conduct of the study.

If a patient or his/her legal representative is not able to read the ICF, a reliable, independent witness should attend the entire process of ICF discussion. The selection of witness must guarantee the patient’s right to confidentiality. The reliable, independent witness should be an independent individual instead of personnel affiliated to the study institution or participating in the study. Family members or acquaintances are good choice for independent witness. If possible, after the subject or patient or his/her legal representative gives verbal consent and signs the ICF, the witness should sign and date the ICF in person to prove the information is correct, since only when the patient or his/her legal representative fully understands the contents of the ICF can the ICF be authentic.

**4. Ethics Committee**

Before the initiation of the study, the study protocol and related documents (e.g., patient information, ICF, Investigator’s Brochure, etc.) should be submitted together to the Ethics Committee for approval. Study documents accepted/approved by the Ethics Committee should be archived. The study can only be conducted after a written approval is obtained.

Any amendment to the study protocol should be submitted to the Ethics Committee, and SAEs should be reported to IRB according to international and/or local requirements.

# XIII. Study Period

The trial is reviewed and approved by the Ethics Committed in May 2019 and is initiated in May 2019;

The trial will be ended in May 2022.

# XIV. Study Personnel

Study Site: Peking University Cancer Hospital and Institute

Study Director: Shen, Lin, Prof.

Contact Number: 010-88196088

# XV. Annexes

Annex 1 ECOG Performance Status Score Scale

Annex 2 RECIST Response Evaluation Criteria in Solid Tumors (RECIST) Version 1.1 (excerpt)

Annex 3 Excerpt of NCI CTCAE Version 4 (CTCAE v4.0, 6/14/2010)

Annex 4 EGFR Immunohistochemistry Scoring Criteria, EGFR Amplification Positive Criteria

References

1. Global Burden of Disease Cancer, C., et al., *Global, Regional, and National Cancer Incidence, Mortality, Years of Life Lost, Years Lived With Disability, and Disability-Adjusted Life-Years for 29 Cancer Groups, 1990 to 2016: A Systematic Analysis for the Global Burden of Disease Study.* JAMA Oncol, 2018. **4**(11): p. 1553-1568.

2. Zhang, X., et al., *A phase II trial of paclitaxel and cisplatin in patients with advanced squamous-cell carcinoma of the esophagus.* Am J Clin Oncol, 2008. **31**(1): p. 29-33.

3. Burkart, C., et al., *A phase II trial of weekly irinotecan in cisplatin-refractory esophageal cancer.* Anticancer Res, 2007. **27**(4C): p. 2845-8.

4. Ettinger, D.S., et al., *Non-Small Cell Lung Cancer, Version 5.2017, NCCN Clinical Practice Guidelines in Oncology.* J Natl Compr Canc Netw, 2017. **15**(4): p. 504-535.

5. Sequist, L.V., et al., *Phase III study of afatinib or cisplatin plus pemetrexed in patients with metastatic lung adenocarcinoma with EGFR mutations.* J Clin Oncol, 2013. **31**(27): p. 3327-34.

6. Wu, Y.L., et al., *Afatinib versus cisplatin plus gemcitabine for first-line therapy of Asian patients with advanced non-small-cell lung cancer harbouring EGFR mutations (LUX-Lung 6): an open-label, randomised phase 3 trial.* Lancet Oncol, 2014. **15**(2): p. 213-22.

7. Park, K., et al., *Afatinib versus gefitinib as first-line therapy of patients with EGFR mutation-positive non-small-cell lung cancer (LUX-Lung 7): a phase 2B, open-label, randomised controlled trial.* Lancet Oncol, 2016. **17**(5): p. 577-89.

8. Jia, J., et al., *The relation of EGFR expression by immunohistochemical staining and clinical response of combination treatment of nimotuzumab and chemotherapy in esophageal squamous cell carcinoma.* Clin Transl Oncol, 2016. **18**(6): p. 592-8.

9. Wang, X., et al., *Predictive value of EGFR overexpression and gene amplification on icotinib efficacy in patients with advanced esophageal squamous cell carcinoma.* Oncotarget, 2016. **7**(17): p. 24744-51.

10. Hanawa, M., et al., *EGFR protein overexpression and gene amplification in squamous cell carcinomas of the esophagus.* Int J Cancer, 2006. **118**(5): p. 1173-80.

11. Kato, H., et al., *Gene amplification of EGFR, HER2, FGFR2 and MET in esophageal squamous cell carcinoma.* Int J Oncol, 2013. **42**(4): p. 1151-8.

12. Petty, R.D., et al., *Gefitinib and EGFR Gene Copy Number Aberrations in Esophageal Cancer.* J Clin Oncol, 2017. **35**(20): p. 2279-2287.

13. Janmaat, M.L., et al., *Predictive factors for outcome in a phase II study of gefitinib in the second-line therapy of advanced esophageal cancer patients.* J Clin Oncol, 2006. **24**(10): p. 1612-9.

14. Liu, Z., et al., *Mouse avatar models of esophageal squamous cell carcinoma proved the potential for EGFR-TKI afatinib and uncovered Src family kinases involved in acquired resistance.* J Hematol Oncol, 2018. **11**(1): p. 109.

**Annex 1 Performance Status Score**

ECOG-Zubrod-WHO Performance Status Score Scale

| Grade | Performance Status | Equivalent to Karnofsky Performance Status |
| --- | --- | --- |
| 0 | Fully active, able to carry on all pre-disease performance without restriction. | 90-100 |
| 1 | Restricted in physically strenuous activity but ambulatory and able to carry out work of a light or sedentary nature, e.g., light house work, office work. | 70-80 |
| 2 | Tolerable to tumor symptoms, ambulatory and capable of all selfcare but unable to carry out any work activities; up and about more than 50% of waking hours | 50-60 |
| 3 | Severe symptoms of tumor; confined to bed or chair more than 50% of waking hours, but able to get out of bed and stand, capable of only limited selfcare | 30-40 |
| 4 | Totally confined to bed or chair; cannot carry on any selfcare | 10-20 |
| 5 | Dead | 0 |

**Annex 2 Response Evaluation Criteria in Solid Tumors (RECIST)**

Definitions

Measurable disease – the presence of at least one measurable lesion. If the measurable disease is restricted to a solitary lesion, its neoplastic nature should be confirmed by cytology/histology.

Measurable lesion – lesions that can be accurately measured in at least one dimension with longest diameter ≥20 mm using conventional techniques or ≥10 mm by spiral CT scan.

Non-measurable lesions – all other lesions, including small lesions (longest diameter <20 mm with conventional techniques or <10 mm with spiral CT scan), i.e., bone lesions, leptomeningeal disease, ascites, pleural/pericardial effusion, inflammatory breast disease, lymphangitis cutis/pulmonis, cystic lesions, and also abdominal masses that are not confirmed and followed by imaging techniques.

- All measurements should be taken and recorded in metric notation, using a ruler or calipers. All baseline evaluations should be performed as closely as possible to the beginning of treatment and never more than 4 weeks before the beginning of treatment.
- The same method of assessment and the same technique should be used to characterize each identified and reported lesion at baseline and during follow-up.
- Clinical lesions will only be considered measurable when they are superficial (e.g., skin nodules and palpable lymph nodes). For the case of skin lesions, documentation by color photography, including a ruler to estimate the size of the lesion, is recommended.

Methods of measurement

- CT and MRI are the best currently available and reproducible methods to measure target lesions selected for response assessment. Conventional CT and MRI should be performed with cuts of 10 mm or less in slice thickness contiguously. Spiral CT should be performed using a 5 mm contiguous reconstruction algorithm. This applies to tumors of the chest, abdomen and pelvis. Head and neck tumors and those of extremities usually require specific protocols.
- Lesions on chest X-ray are acceptable as measurable lesions when they are clearly defined and surrounded by aerated lung. However, CT is preferable.
- When the primary endpoint of the study is objective response evaluation, ultrasound (US) should not be used to measure tumor lesions. It is, however, a possible alternative to clinical measurements of superficial palpable lymph nodes, subcutaneous lesions and thyroid nodules. US might also be useful to confirm the complete disappearance of superficial lesions usually assessed by clinical examination.
- The utilization of endoscopy and laparoscopy for objective tumor evaluation has not yet been fully and widely validated. Their uses in this specific context require sophisticated equipment and a high level of expertise that may only be available in some centers. Therefore, the utilization of such techniques for objective tumor response should be restricted to validation purposes in specialized centers. However, such techniques can be useful in confirming complete pathological response when biopsies are obtained.
- Tumor markers alone cannot be used to assess response. If markers are initially above the upper normal limit, they must normalize for a patient to be considered in complete clinical response when all lesions have disappeared.
- Cytology and histology can be used to differentiate between PR and CR in rare cases (e.g., after treatment to differentiate between residual benign lesions and residual malignant lesions in tumor types such as germ cell tumors).

Baseline documentation of “target” and “non-target” lesions

- All measurable lesions up to a maximum of 5 lesions per organ and 10 lesions in total, representative of all involved organs should be identified as target lesions and recorded and measured at baseline.
- Target lesions should be selected on the basis of their size (lesions with the longest diameter) and their suitability for accurate repeated measurements (either by imaging techniques or clinically).
- A sum of the longest diameter (LD) for all target lesions will be calculated and reported as the baseline sum LD. The baseline sum LD will be used as reference by which to characterize the objective tumor response.
- All other lesions (or sites of disease) should be identified as non-target lesions and should also be recorded at baseline. Measurements of these lesions are not required, but the presence or absence of each should be noted throughout follow-up.

Response criteria

| Evaluation of target lesion |  |
| --- | --- |
| Complete response (CR): | Disappearance of all target lesions. |
| Partial response (PR): | At least a 30% decrease in the sum of the LD of target lesions, taking as reference the baseline sum LD. |
| Progressive disease (PD): | At least a 20% increase in the sum of the LD of target lesions, taking as reference the smallest sum LD recorded since the treatment started or the appearance of one or more new lesions. |
| Stable disease (SD): | Neither sufficient shrinkage to qualify for PR nor sufficient increase to qualify for PD, taking as reference the smallest sum LD since the treatment started. |

Evaluation of non-target lesions

| Complete response (CR): | Disappearance of all non-target lesions and normalization of tumor marker level. |
| --- | --- |
| Incomplete response or stable disease (SD): | Persistence of one or more non-target lesion(s) or/and maintenance of tumor marker level above the normal limits. |
| Progressive disease (PD): | Appearance of one or more new lesions and/or unequivocal progression of existing non-target lesions. Although an unequivocal progression of existing “non-target” lesions is exceptional, in such circumstances, the opinion of the treating physician should prevail and the progression status should be confirmed later on by the review panel (or study chair). |

Evaluation of best overall response

The best overall response is the best response recorded from the start of the treatment until disease progression/recurrence (taking as reference for PD the smallest measurements recorded since the treatment started). In general, the patient’s best response assignment will depend on the achievement of both measurement and confirmation criteria.

| Target lesions | Non-target lesions | New lesions | Overall response |
| --- | --- | --- | --- |
| CR | CR | No | CR |
| CR | Non-CR/SD | No | PR |
| PR | Non-PD | No | PR |
| SD | Non-PD | No | SD |
| PD | Any | Yes or No | PD |
| Any | PD | Yes or No | PD |
| Any | Any | Yes | PD |

- Patients with a global deterioration of health status requiring discontinuation of treatment without objective evidence of disease progression at that time should be classified as having “symptomatic deterioration”. Every effort should be made to document the objective progression even after discontinuation of treatment.
- In some circumstances it may be difficult to distinguish residual disease from normal tissue. When the evaluation of complete response depends on this determination, it is recommended that the residual lesion be investigated (fine needle aspirate/biopsy) to confirm the complete response status.
- Confirmation.
- The main goal of confirmation of objective response is to avoid overestimating the response rate observed. In cases where confirmation of response is not feasible, it should be made clear when reporting the outcome of such studies that the responses are not confirmed.
- To be assigned a status of PR or CR, changes in tumor measurements must be confirmed by repeat assessments that should be performed no less than 4 weeks after the criteria for response are first met. Longer intervals as determined by the study protocol may also be appropriate.
- In the case of SD, follow-up measurements must have met the SD criteria at least once after study entry at a minimum interval (in general, not less than 6-8 weeks) that is defined in the study protocol.
- Duration of overall response.
- The duration of overall response is measured from the time measurement criteria are met for CR or PR (whichever status is recorded first) until the first date that recurrence or PD is objectively documented, taking as reference for PD the smallest measurements recorded since the treatment started.
- Duration of stable disease
- SD is measured from the start of the treatment until the criteria for disease progression are met, taking as reference the smallest measurements recorded since the treatment started.
- The clinical relevance of the duration of SD varies for different tumor types and grades. Therefore, it is highly recommended that the protocol specifies the minimal time interval required between two measurements for determination of SD. This time interval should take into account the expected clinical benefit that such a status may bring to the population under study.
- Response review.
- For clinical trials where the response rate is the primary endpoint it is strongly recommended that all responses be reviewed by an expert(s) independent of the study at the study’s completion. Simultaneous review of the patients’ files and radiological images is the best approach.
- Reporting of results.
- All patients included in the study must be assessed for response to treatment, even if there are major trial protocol treatment deviations or if they are ineligible. Each patient will be assigned one of the following categories: 1) complete response, 2) partial response, 3) stable disease, 4) progressive disease, 5) early death from malignant disease, 6) early death from toxicity, 7) early death because of other cause, or 9) unknown (not assessable, insufficient data).
- All of the patients who met the eligibility criteria should be included in the main analysis of the response rate. Patients in response categories 4-9 should be considered as failing to respond to treatment (disease progression). Thus, an incorrect treatment schedule or drug administration does not result in exclusion from the analysis of the response rate. Precise definitions for categories 4-9 will be protocol specific.
- All conclusions should be based on all eligible patients.
- Sub-analyses may then be performed on the basis of a subset of patients, excluding those for whom major protocol deviations have been identified (e.g., early death due to other reasons, early discontinuation of treatment, major protocol violations, etc.). However, these sub-analyses may not serve as the basis for drawing conclusions concerning treatment efficacy, and the reasons for excluding patients from the analysis should be clearly reported.
- The 95% confidence intervals should be provided.

**Annex 3 Common Terminology Criteria for Adverse Events v4.0 (CTCAE)**

The descriptions and grading scale in NCI Common Terminology Criteria for Adverse Events (CTCAE) Version 4.0 will be used for reporting of AE (<https://ctep.cancer.gov/protocolDevelopment/electronic_applications/ctc.htm>).

**Annex 4 EGFR Immunohistochemistry Scoring Criteria, EGFR Amplification Positive Criteria**

- - - 1. EGFR immunochemistry scoring criteria: Immunohistochemistry (IHC) was carried out according to the manufacturer’s instructions to assess the expression of EGFR (Clone E30, DAKO, Glostrup, Denmark; 1:50). Positive staining was deﬁned as any membrane staining above background level (deﬁned as the level noted in a negative control sample) in ≥ 10% cancer cells of any intensity, with 1+ equating to faint or barely perceptible membrane staining, 2+ indicating weak to moderate staining of the complete cell membrane, and 3+ indicating strong staining of the complete cell membrane. The staining was evaluated by two independent, blinded investigators.
      2. EGFR amplification: (1) in tumor cells, ratio of EGFR gene copy number to CEP7 copy number ≥2; (2) presence of EGFR gene clusters in ≥10% of the tumor cells; (3) ≥15 EGFR gene copies in ≥10% of the tumor cells.
